# *OsUGE2* Regulates Plant Growth through Affecting ROS Homeostasis and Iron Level in Rice

**DOI:** 10.1186/s12284-024-00685-0

**Published:** 2024-01-12

**Authors:** Shuaiqi Yang, Nana Chen, Jiaxuan Qi, Abdul Salam, Ali Raza Khan, Wardah Azhar, Chunyan Yang, Nuo Xu, Junyu Wu, Yihua Liu, Bohan Liu, Yinbo Gan

**Affiliations:** 1 Zhejiang Key Lab of Crop Germplasm, Department of Agronomy, College of Agriculture and Biotechnology, Zhejiang University, Hangzhou, 310000 China; 2 College of Agriculture and Forestry Sciences, Linyi University, Linyi, 276000 Shandong China; 3 College of Agriculture, Hunan Agricultural University, Changsha, 410128 China

**Keywords:** *Oryza sativa* L., OsUGE2, UDP-Glucose/Galactose, Growth, Reactive oxygen species, Iron

## Abstract

The growth and development of rice (*Oryza sativa *L.) are affected by multiple factors, such as ROS homeostasis and utilization of iron. Here, we demonstrate that *OsUGE2*, a gene encoding a UDP-glucose 4-epimerase, controls growth and development by regulating reactive oxygen species (ROS) and iron (Fe) level in rice. Knockout of this gene resulted in impaired growth, such as dwarf phenotype, weakened root growth and pale yellow leaves. Biochemical analysis showed that loss of function of *OsUGE2* significantly altered the proportion and content of UDP-Glucose (UDP-Glc) and UDP-Galactose (UDP-Gal). Cellular observation indicates that the impaired growth may result from decreased cell length. More importantly, RNA-sequencing analysis showed that knockout of *OsUGE2* significantly influenced the expression of genes related to oxidoreductase process and iron ion homeostasis. Consistently, the content of ROS and Fe are significantly decreased in *OsUGE2* knockout mutant. Furthermore, knockout mutants of OsUGE2 are insensitive to both Fe deficiency and hydrogen peroxide (H_2_O_2_) treatment, which further confirmed that *OsUGE2* control rice growth possibly through Fe and H_2_O_2_ signal. Collectively, these results reveal a new pathway that *OsUGE2* could affect growth and development via influencing ROS homeostasis and Fe level in rice.

**Supplementary Information:**

The online version contains supplementary material available at 10.1186/s12284-024-00685-0.

## Background

Rice is the most widely cultivated cereal crop all around the world (Jiang et al. 2017). It is well known that multiple factors could affect the growth and development of rice, such as intrinsic genetic information, plant hormones, signaling molecule, nutritional status and external environmental factors, including light, temperature and water, etc. (Araki et al. 2015; Mittler 2017; Shahid and Abdul 2020; Wang et al. 2022a). The integration of the above factors finally determines the performance of growth and development in rice.

Reactive oxygen species (ROS), which can act as a signal molecule, is thought to be as byproducts of aerobic metabolism with different forms, including singlet oxygen, superoxide, hydrogen peroxide and hydroxyl radical in plants (Raymond and Segrè, 2006; Schmidt and Schippers 2015), which are mainly generated in chloroplasts, peroxisomes and mitochondria (Asada et al. 1974; Xu et al. 2009; Huang et al. 2016). So far, the roles of ROS in plants have been extensively studied. It was found that ROS can regulate growth and development, stress signaling, systemic response and cell death (Foreman et al. 2003; Mittler 2017). Excessive ROS can be toxic to plants, mainly through damaging DNA, RNA, protein and membrane (Mittler et al. 2004; Foyer and Noctor 2013; Vaahtera et al. 2014). However, basal ROS are beneficial to plants, which are essential for cellular proliferation, differentiation, physiological function and viability (Lu et al. 2014; Schmidt and Schippers 2015; Mittler 2017). To maintain a suitable ROS level in plants, two types of ROS scavenging mechanisms have been evolved under normal or stress circumstances, which are enzymatic antioxidant defense system and non-enzymatic antioxidant defense system, respectively. The enzymatic systems exert ROS scavenging function mainly through superoxide dismutase (SOD), catalase (CAT), ascorbate peroxidase (APX) and glutathione peroxidase (GPX) (Huang et al. 2019). The latter system is mainly relied on low molecular mass antioxidants, such as flavonoids and glutathione (Gechev et al. 2006). In plants, aberrant level of ROS can have obviously effects on the growth and development. It was reported that a transcription factor KUODA1 (KUA1), controls cell expansion during leaf development in *Arabidopsis thaliana*, mainly through repressing the expression of a set of genes encoding peroxidases that regulate ROS homeostasis in the apoplast, and disruption of *KUA1* causes decreased ROS level and smaller leaf cells (Lu et al. 2014). HOMOLOG OF BRASSINOSTEROID ENHANCED EXPRESSION2 INTERACTING WITH IBH (HBI) transcription factor positively regulates the expression of a series of antioxidant genes to reduce the ROS accumulation in *Arabidopsis thaliana*, and loss of function of HBI leads to high level of H_2_O_2_, which impairs the nuclear localization of NLP7, further repressing plant growth and development (Chu et al. 2021). OsWOX11, a WUSHEL-RALATED homeobox (WOX) family transcription factor, upregulating peroxidase genes in crown root meristem, could promote lysine acetylation of nitrogen metabolism and peptide/protein synthesis related proteins, further promoting the growth of crown roots (Xu et al. 2022). Moreover, overexpression of *OsbZIP23* enhances rice seed vigor, mainly by directly promoting the expression of *Oryza Sativa 1-CYS PEROXIREDOXIN 1A* (*OsPER1A*), a key gene in the detoxification pathway. Further studies showed that the enhanced seed vigor was possible due to decreased H_2_O_2_ content (Wang et al. 2022b). Therefore, maintaining a suitable ROS level is crucial for normal growth and development in plants (Mittler 2017; Huang et al. 2019).

Iron is one of the essential nutrients for plant growth and development (Lee and An 2009). In plants, Fe is involved in some key metabolic processes, such as redox reactions, photosynthesis, chloroplast development, chlorophyII biosynthesis and respiration (Marschner 1995; Kobayashi and Nishizawa 2012). Iron deficiency in plants leads to chlorosis and decreased biomass, crop yield and quality (Takahashi et al. 2001; Yang et al. 2013). Plants have evolved two different strategies to uptake Fe from the rhizosphere, the reduction strategy (Strategy I) for non-graminaceous plants and the chelation strategy for graminaceous (Strategy II) (Mori 1999). For strategy I, plant roots excrete protons to the rhizosphere to reduce the pH to increase the solubility of Fe (III), and then Fe (III) is reduced to Fe (II) by FERRIC REDUCTASE OXIDASES (FROs), further imported into the root cells by IRON REGULATED TRANSPORTERs (IRTs) (Eide et al. 1996; Robinson et al. 1999; Santi and Schmidt 2009; Wang et al. 2020). For strategy II, plants synthesize and secrete mugineic acid (MA) family phytosiderophores (PSs) in the root to chelate Fe (III) (Römheld and Marschner 1986). Phytosiderophores are synthesized from S-adenosyl-methionine through a set of sequential enzymatic reactions, including NICOTIAN AMINE SYNTHASE (NAS), NICOTIANAMINE AMINOTRANFERASE (NAAT) and DEOXYMUGINEIC ACID SYNTHASE (DMAS) (Higuchi et al. 1999; Takahashi et al. 1999; Bashir et al. 2006). Then PSs are exported to the rhizosphere by TRANSPROTER OF MUGINEIC ACID FAMILY PHYTOSIDEROPHORESs (TOMs) (Nozoye et al. 2011, 2015). Finally, the PSs and Fe (III) form a complex and imported into root cells by YELLOW STRIPE 1 (YS1) or YS1-LIKE (YSL) (Curie et al. 2001; Murata et al. 2006; Inoue et al. 2009). Interestingly, rice utilizes both strategy II and partial strategy I (Kawakami and Bhullar 2018; Li et al. 2020). In addition, several transcription factors have been identified to participate in Fe homeostasis, including OsIDEF1 (Kobayashi et al. 2007), OsIDEF2 (Ogo et al. 2008), OsIRO2 (Ogo et al. 2007), OsIRO3 (Zheng et al. 2010), OsbHLH133 (Wang et al. 2013), OsbHLH60 (Zhang et al. 2017), OsbHLH156 (Wang et al. 2019), OsbHLH058 and OsbHLH059 (Kobayashi et al. 2019) and OsbHLH061 (Wang et al. 2022c).

There are four UDP-glucose 4-epimerases (UGEs) in rice, named OsUGE1, OsUGE2, OsUGE3 and OsUGE4, which have the catalysis ability converting UDP-glucose into UDP-galactose and UDP-galactose into UDP-glucose (Liu et al. 2007; Kim et al. 2009). *OsUGE1* was demonstrated to control pollen fertility by promoting the tapetum degradation (Wang et al. 2023; Chen et al. 2023). *OsUGE3* was reported to increase biomass production, mechanical strength and salt stress tolerance by modification of cell wall (Tang et al. 2022). The *Osuge2* mutants, also named *Osfc24* or *Osbp1* mutants, showed a brittleness phenotype (Zhang et al. 2020a, b). The cell wall composition of *Osfc24* mutant was significantly changed and the orientation of cellulose microfibrils was disrupted (Zhang et al. 2020a). Similarly, *Osbp1* mutant also showed altered sugar composition and structure of cell wall (Zhang et al. 2020b). However, the mechanism that *OsUGE2* controls rice growth and development still remains largely unclear.

In this study, we showed that the loss of function of *OsUGE2* could significantly retard growth and development, at least partly by decreasing ROS and Fe level in rice. As a UDP-galactose/glucose epimerase, the ratio and content of UDP-Glc and UDP-Gal were remarkably altered in knockout mutants of *OsUGE2*. Moreover, the functional deficiency of OsUGE2 significantly affect the expression of genes relevant to oxidoreductase process and iron ion homeostasis, finally resulting in reduced ROS and Fe level. Collectively, our results provide a novel mechanism of *OsUGE2* controlling rice growth.

## Results

### Knockout of *OsUGE2* Impairs Rice Growth

**Fig. 1:**
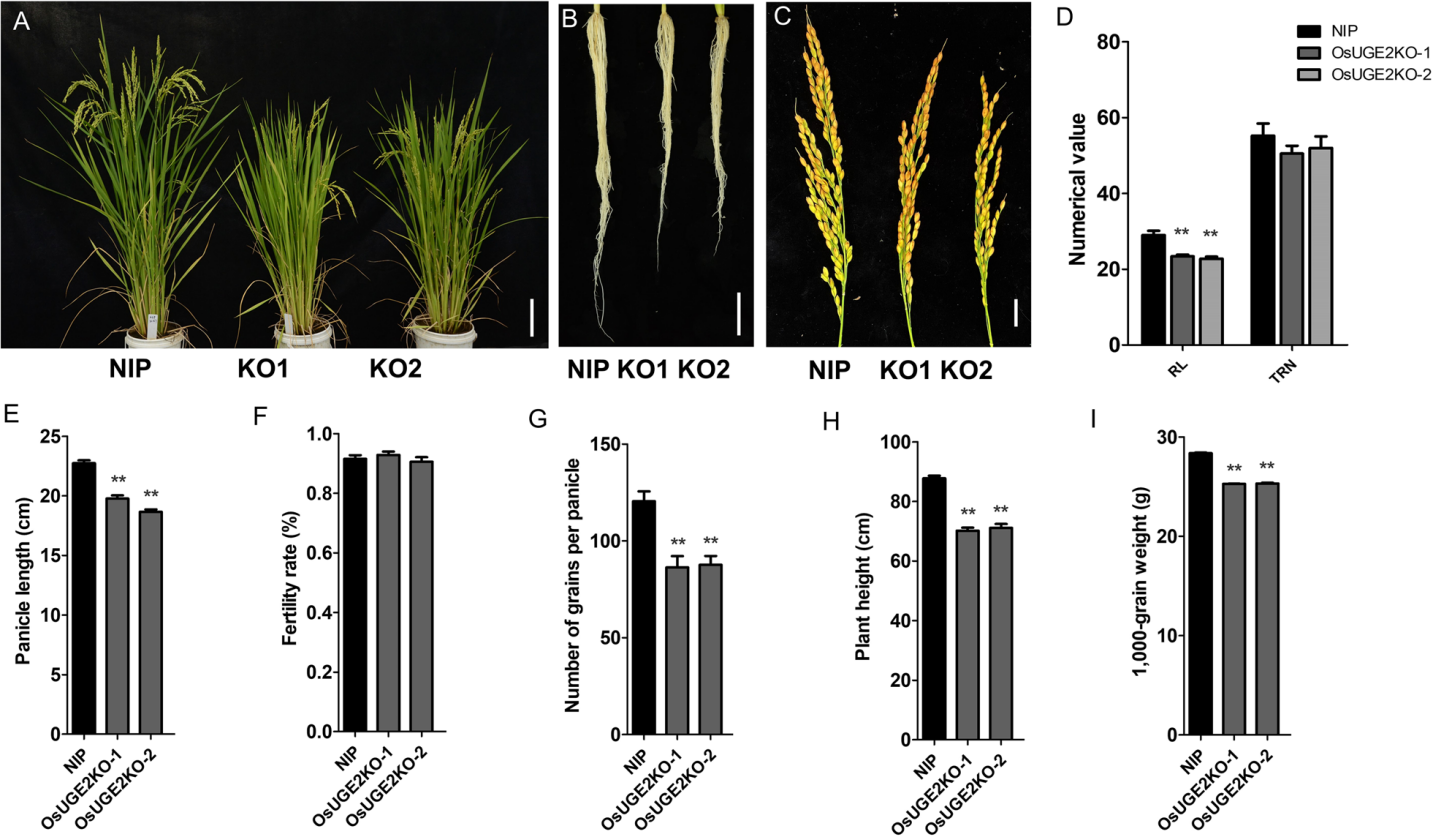
Knockout of *OsUGE2* weakened growth and development of rice. **A** Morphology of NIP, *OsUGE2KO-1*(KO-1) and *OsUGE2KO-2*(KO-2) at mature stage. Bars = 10 cm. **B** Root morphology of 2-month-old NIP, KO1 and KO2 cultivated with normal YNS hydroponic culture. Bars = 5 cm. **C** Mature panicle morphology of NIP, KO1 and KO2. Bars = 2 cm. **D** Statistical analysis of root length (RL) and total root number (TRN) of 2-month-old NIP, KO1 and KO2 cultivated with normal YNS hydroponic culture. The unit for RL is centimeter (cm). At least 12 plants were measured for each genotype. **E**–**H** Statistical analysis of panicle length (**E**), fertility rate (**F**), number of grains per panicle (**G**) and plant height (**H**) of NIP, KO1 and KO2. At least 12 measured values for each trait were acquired. **I** Statistical analysis of 1,000-grain weight of NIP, KO1 and KO2. Three replications were used. Data were means ± SEM. Asterisks indicated significant differences according to Student’s *t* test (*P* ≤ 0.05**)

Previous studies showed that *UGEs* in *Arabidopsis thaliana* play important roles in growth and development (Rösti et al. 2007). We therefore created mutants of *OsUGE2* through CRISPR-Cas9 technology (Ma and Liu 2016). To ensure complete knockout the function of *OsUGE2*, the target sequence was designed in front of the NAD binding site, which was upstream of active site, substrate site and homodimer interface (Additional file 1: Fig. S1). Two independent frame shift mutant lines were selected for further analysis (Additional file 2: Fig. S2). It was found that at grain filling stage, compared with wild type (NIP), the *OsUGE2* knockout mutants displayed some obvious morphological abnormalities, such as reduced plant height, pale yellow leaves (Fig. 1A, H). By using hydroponic method, we also observed decreased root length and number of *OsUGE2* knockout mutants compared with NIP at 2 months old age, although the difference of total root number was not significant (Fig. 1B, D). Furthermore, the length of panicle was also significantly reduced, accompanied with less grains per panicle and 1,000-grain weight (Fig. 1C, E, G, I). Unlike the previous report that described impaired fertility rate in *Arabidopsis thaliana uges* mutants (Rösti et al. 2007), it seems that the pollen fertility in *OsUGE2* knockout mutants was not affected due to the similar fertility rates compared with NIP (Fig. 1F). Even more, we also generated quadruple mutants (simultaneous mutation of *OsUGE1*, *OsUGE2*, *OsUGE3* and *OsUGE4*) by using CRISPR-Cas9 method, and found that at tillering stage, the growth was severely affected (Additional file 3: Fig. S3). In addition, loss of function of *OsUGE2* significantly increased the transcript level of *OsUGE3* and *OsUGE4* (Additional file 4: Fig. S4), while the phenotype of *OsUGE2* knockout mutant was not restored, which indicates that the functional specificity of *OsUGE* gene family. Overall, these results clearly indicated that *OsUGE2* and the other *OsUGE* family genes (*OsUGE1*, *OsUGE3* and *OsUGE4*) are crucial for normal growth and development in rice.

### The Cell Length is Decreased in *OsUGE2* Knockout Mutants

**Fig. 2:**
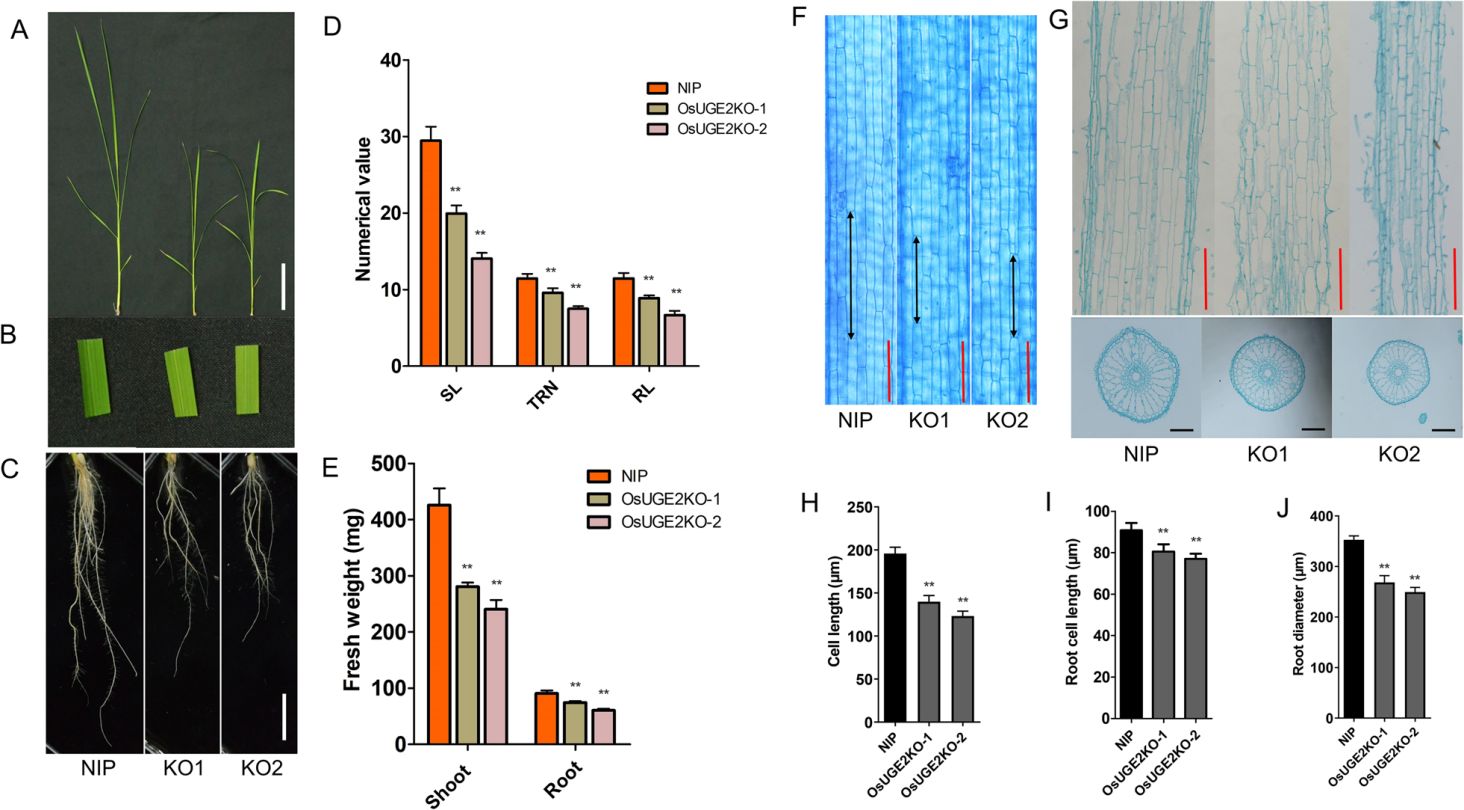
Loss of function of *OsUGE2* decreases the cell length. **A** Shoot morphology of NIP, KO1 and KO2 grown in normal YNS hydroponic culture for 18 days. Bars = 5 cm. **B** Display of leaf color of NIP, KO1 and KO2 grown in normal YNS hydroponic culture for 18 days. **C** Root morphology of NIP, KO1 and KO2 grown in normal YNS hydroponic culture for 18 days. Bars = 2 cm. **D** Statistical analysis of shoot length (SL), total root number (TRN) and root length (RL) in NIP, KO1 and KO2 grown in normal YNS hydroponic culture for 18 days. The unit for SL and RL were centimeter (cm). At least 12 plants were measured for each genotype. **E** Statistical analysis of fresh weight of shoot and root in NIP, KO1 and KO2 cultivated in normal YNS hydroponic culture for 18 days. At least 12 plants were measured. **F** The cell length of inner-epidermal cells of leaf sheaths staining with 0.5% toluidine blue. The double arrow indicated the cell length. 3-week-old seedlings grown in normal YNS hydroponic culture were used. Bars = 100 μm. **G** The longitudinal (up) and cross sections (down) of roots with 3-week-old seedlings grown in normal YNS hydroponic culture. Bars = 100 μm. **H**–**J** Statistical analysis of cell length of inner-epidermal cells of leaf sheaths (**H**), Root cell length (**I**) and root diameter (**J**). Six plants were used for each genotype. Data were means ± SEM. Asterisks indicated significant differences according to Student’s *t* test (*P* ≤ 0.05**)

To analyze the effect of *OsUGE2* on growth in detail, 20-day-old seedlings cultivated by hydroponic method were used for investigation. As shown in Fig. 2A, C, D, the shoot length (SL), total root number (TRN) and root length (RL) were significantly reduced compared with those of NIP. Similarly, the fresh weight of shoots and roots were also significantly decreased in *OsUGE2* knockout mutants (Fig. 2E). In addition, the leaves of *OsUGE2* knockout mutant presented pale yellow color, similar with that at grain-filled stage (Figs. 1A, 2B), which suggested that the pigment should be aberrant. Indeed, compared with NIP, the chlorophyll a and chlorophyll b were notably decreased, including the content of chlorophyll a plus chlorophyll b (Additional file 5: Fig. S5A). Meanwhile, the photosynthetic ability was also significantly weakened (Additional file 5: Fig. S5B to E). Moreover, in order to explore the alteration of growth at cellular level, inner-epidermal of leaf sheath and sections of root were used for observation. Both the leaf sheaths and roots were stained with toluidine blue and the cross and longitudinal sections were obtained by paraffin section method. It was shown that the cell length of inner-epidermal cells of leaf sheaths and roots were remarkably reduced referring to NIP (Fig. 2F–I). For the cross section of roots, it was obvious that the root diameter decreased compared to that of NIP, and the reduced diameter seems to be caused by decreased area of air chamber in roots (Fig. 2J). Collectively, the small-size phenotype of *OsUGE2* mutant was mainly caused by reduced cell length.

### Loss of Function of *OsUGE2* Alters the Proportion and Content of UDP-Glc and UDP-Gal

**Fig. 3:**
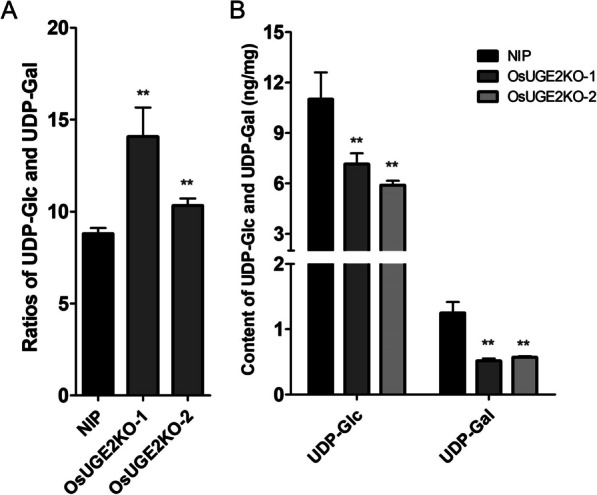
Knockout of *OsUGE2* alters the proportion and content of UDP-Glc and UDP-Gal. **A** The proportion of UDP-Glc/UDP-Gal in NIP, *OsUGE2KO-1* and *OsUGE2KO-2*. **B** The content of UDP-Glc and UDP-Gal in NIP, *OsUGE2KO-1* and *OsUGE2KO-2*. The shoots of 1-week-old seedlings grown under normal YNS hydroponic culture were used for measuring the content of UDP-Glc and UDP-Gal. Four replications were used. Data were means ± SEM. Asterisks indicated significant differences according to Student’s *t* test (*P* ≤ 0.05**)

Previous studies showed that OsUGE2 has UDP-glucose 4-epimerase activity, which could catalyze UDP-glucose converting into UDP-galactose and UDP-galactose into UDP-glucose (Zhang et al. 2020a, b). Meantime, different OsUGEs may have distinct preference of catalysis direction, which was also indicated in *Arabidopsis thaliana* (Barber et al. 2006; Kim et al. 2009). However, all the assessments were performed in vitro. Here, we estimated the catalysis direction preference in vivo, through using *OsUGE2* knockout mutants in rice. To obtain this goal, 7 days old seedlings cultivated with normal hydroponic method were sampled to measure the content of UDP-Glc and UDP-Gal by using Liquid Chromatograph Mass Spectrometer (LC–MS) technology. It was shown that in *OsUGE2* knockout mutant, the ratios of UDP-Glc and UDP-Gal were significantly higher than that of NIP (Fig. 3A), which indicates that OsUGE2 prefers to catalyze UDP-Glc to UDP-Gal. Moreover, the content of UDP-Glc and UDP-Gal were both reduced in *OsUGE2* knockout mutant (Fig. 3B). Overall, these results showed that the homeostasis of UDP-Glc and UDP-Gal was significantly affected in *OsUGE2* knockout mutant.

### Expression Pattern of *OsUGE2*

**Fig. 4:**
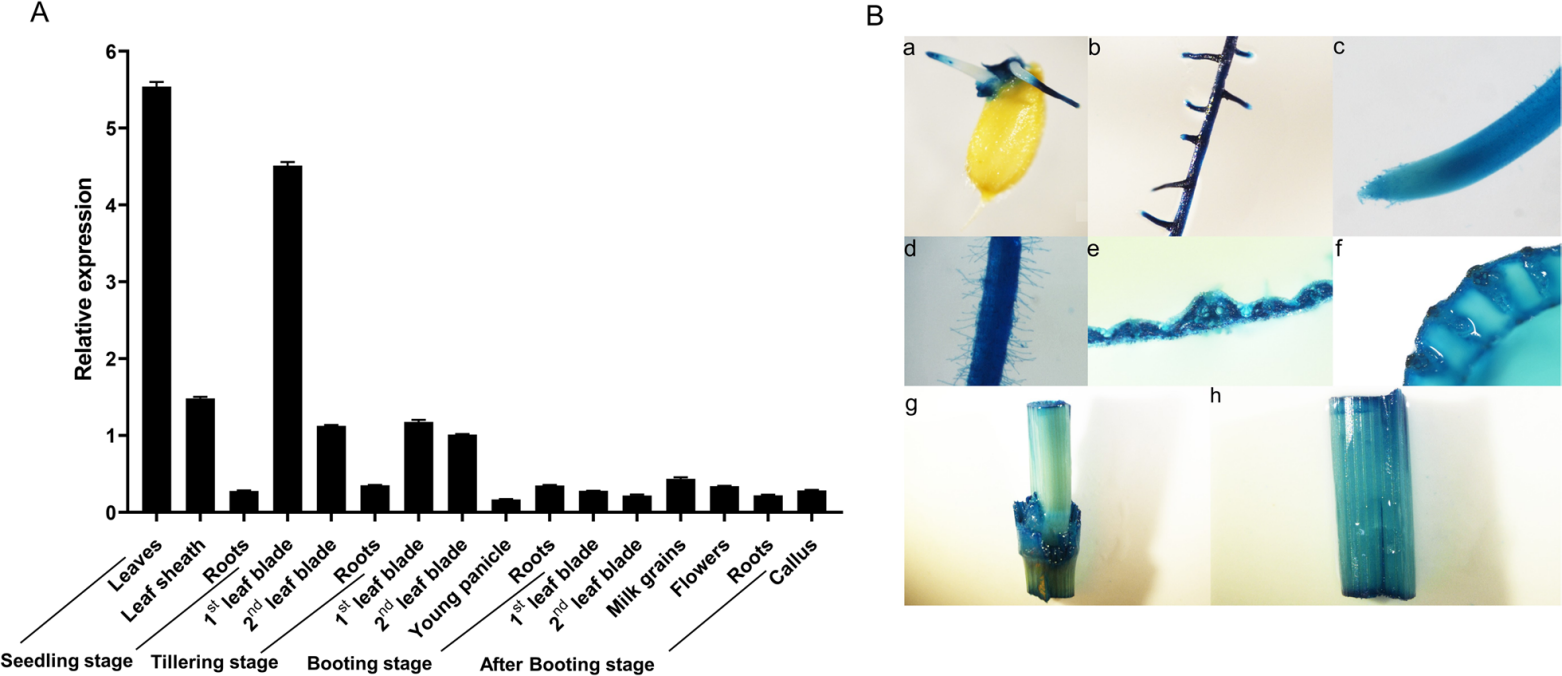
Expression pattern of *OsUGE2*. **A** qRT-PCR analysis of *OsUGE2* expression pattern in various tissues at different stages. *OsActin* gene was used as an internal control. Three biological replications were used. Data were means ± SEM. **B** Detection of GUS activity in *Pro*_*OsUGE2*_*::GUS* transgenic plants. (a) germination seeds; (b) adventitious root; (c) root tip; (d) root hair of primary root; (e) cross section of leaf; (f) cross section of leaf sheath; (g) node; (h) stem

The detailed temporal-spatial expression profile of *OsUGE2* was examined by qRT-PCR and β-glucuronidase (GUS) reporter assay. The qRT-PCR analysis displayed that *OsUGE2* was ubiquitously expressed in all 16 tested tissues (Fig. 4A). Generally, *OsUGE2* dominantly expressed at seedling stage and tillering stage (Fig. 4A). Similarly, *OsUGE2* promoter::GUS reporter analysis revealed that *OsUGE2* was also broadly expressed in various tissues (Fig. 4B). In germination seeds, *OsUGE2* was mainly expressed in tips of buds and seminal roots (Fig. 4B-a). In roots, the protein was significantly accumulated, except for apical parts of lateral roots and adventitious roots (Fig. 4B-b, c and d). Furthermore, it was observed that *OsUGE2* strongly expressed in mesophyll cells, vascular bundle of leaf sheath, node and stem (Fig. 4B-e to h).

### Subcellular Localization of OsUGE2

**Fig. 5:**
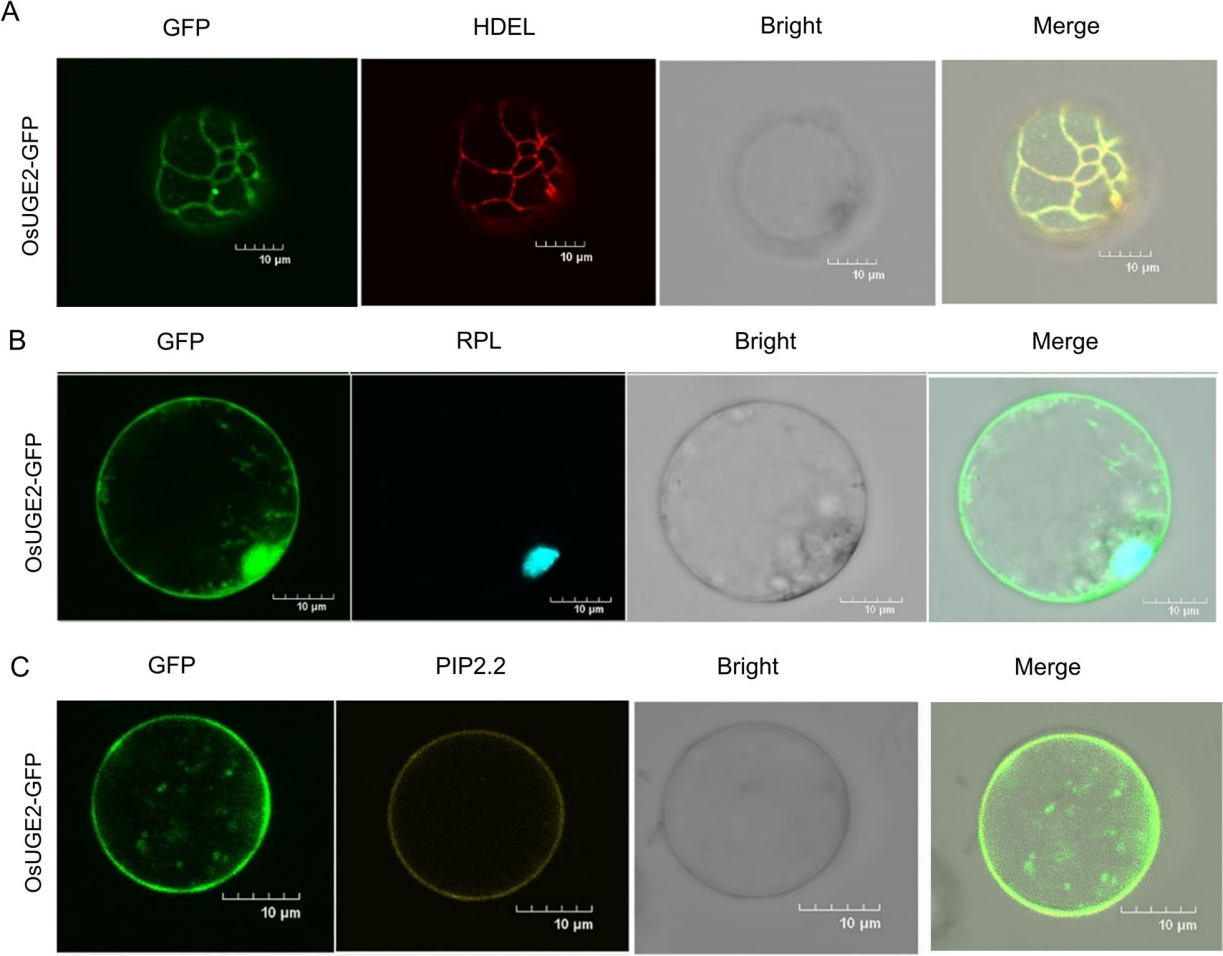
Subcellular localization of OsUGE2 protein in rice protoplasts. **A** Subcellular localization analysis of OsUGE2 protein in endoplasmic reticulum. Endoplasmic reticulum marker HDEL-RFP was as a reference. Scale bars = 10 μm. **B** Subcellular localization analysis of OsUGE2 protein in nucleus. Nucleus marker RPL-CFP was as a reference. Scale bars = 10 μm. **C** Subcellular localization analysis of OsUGE2 protein in plasma membrane. Plasma membrane protein PIP2.2 was as a reference. Scale bars = 10 μm

In order to examine the subcellular localization of OsUGE2 in rice protoplasts, fused expression vector *OsUGE2-GFP* was constructed. The leaf sheaths of 10 days old NIP hydroponic seedlings grown under dark conditions were used for extracting protoplasts. PEG-mediated method was applied to transfer the fused plasmids to protoplasts. Meanwhile, an endoplasmic reticulum marker HDEL-RFP, a nucleus marker RPL-CFP and a plasma membrane localization fused protein PIP2.2-YFP were used to confirm the precisely distribution of OsUGE2 protein. After co-expression in protoplasts, confocal microscopy (FW-SW) was used to observe the localization results. It was observed that OsUGE2 was mainly located on endoplasmic reticulum, cell nucleus and plasma membrane (Fig. 5). The distribution of OsUGE2 on endoplasmic reticulum may indicate that OsUGE2 involved in the sugar metabolism, which was consistent with its enzyme function. It was interesting that OsUGE2 was also located in cell nucleus, which might imply that like OsUGE1 (Wang et al. 2023), OsUGE2 may also act as a transcription factor.

### RNA-seq Analysis of *OsUGE2* Knockout Mutants

**Fig. 6:**
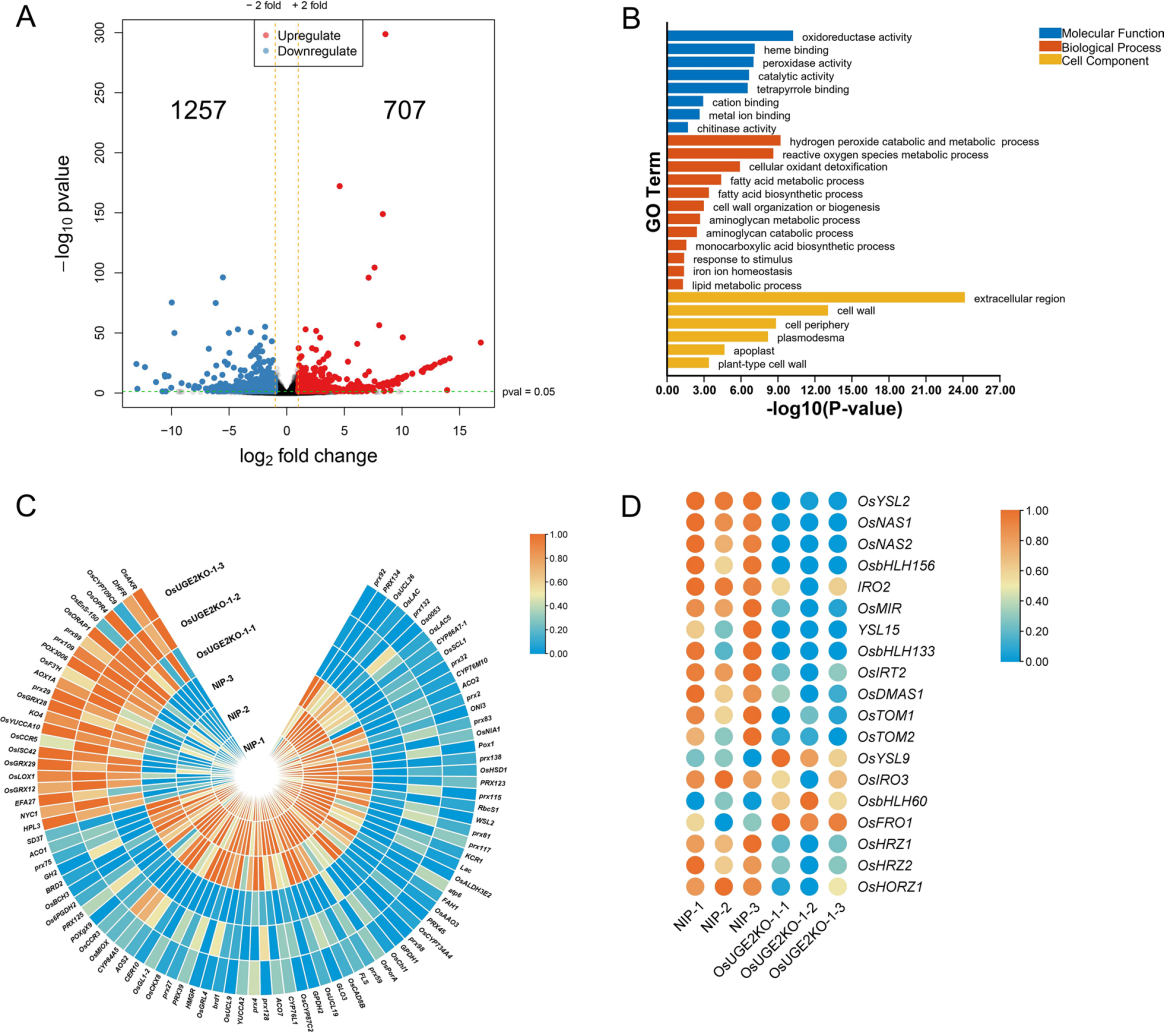
RNA-seq analysis of NIP and *OsUGE2* knockout mutant. **A** Volcano plot of differentially expressed genes (DEGs) between NIP and *OsUGE2* knockout mutant. **B** Functional categorization of the DEGs between NIP and *OsUGE2* knockout mutant based on Gene Ontology. **C** Heatmap of DEGs clustering related to hydrogen peroxide catabolic and metabolic process. **D** Heatmap of DEGs clustering related to Fe homeostasis. Three-week-old seedlings cultivated by normal YNS hydroponic culture were used. Total RNA extracted from the whole rice seedlings of NIP and *OsUGE2KO-1* line was used for RNA-seq analysis with three biological replicates

Considering the obvious alteration of growth in *OsUGE2* knockout mutants, it means that some biological progresses could be abnormal. To figure out which genes were affected, the transcriptomes of NIP and *OsUGE2* knockout mutant were compared via RNA-sequencing (RNA-seq) analysis. Three-week-old seedlings cultivated with hydroponic method were used. Compared with NIP, the results showed that a total of 1964 genes were differentially expressed (≥ 2 folds change at *P* ≤ 0.05; 707 genes were upregulated and 1257 genes were downregulated in *OsUGE2* knockout mutant) (Fig. 6A). GO analysis showed that these differentially expressed genes (DEGs) are involved in various biological processes (Fig. 6B). Of them, hydrogen peroxide catabolic and metabolic process and iron ion homeostasis attracted our special attention, because previous studies identified that ROS and iron play important roles in growth and development of plants (Marschner 1995; Takahashi et al. 2001; Kobayashi and Nishizawa 2012; Yang et al. 2013; Mittler 2017). Further analysis showed that the transcripts levels of genes related to hydrogen peroxide catabolic and metabolic process in *OsUGE2* knockout mutant were significantly altered compared with NIP (Fig. 6C). Similarly, in *OsUGE2* knockout mutant, genes involved in Fe acquisition and transport were also remarkably changed and most of them were downregulated compared to those of NIP (Fig. 6D). As expected, qRT-PCR analysis using *OsUGE2KO-1* and *OsUGE2KO-2* lines displayed similar patterns as the RNA-seq data (Additional file 6: Fig. S6). These results suggest that abnormal expression of genes related to H_2_O_2_ and Fe may cause alteration of ROS homeostasis and Fe level in rice.

### The H_2_O_2_ and Fe Content were Decreased in ***OsUGE2*** Mutants

**Fig. 7:**
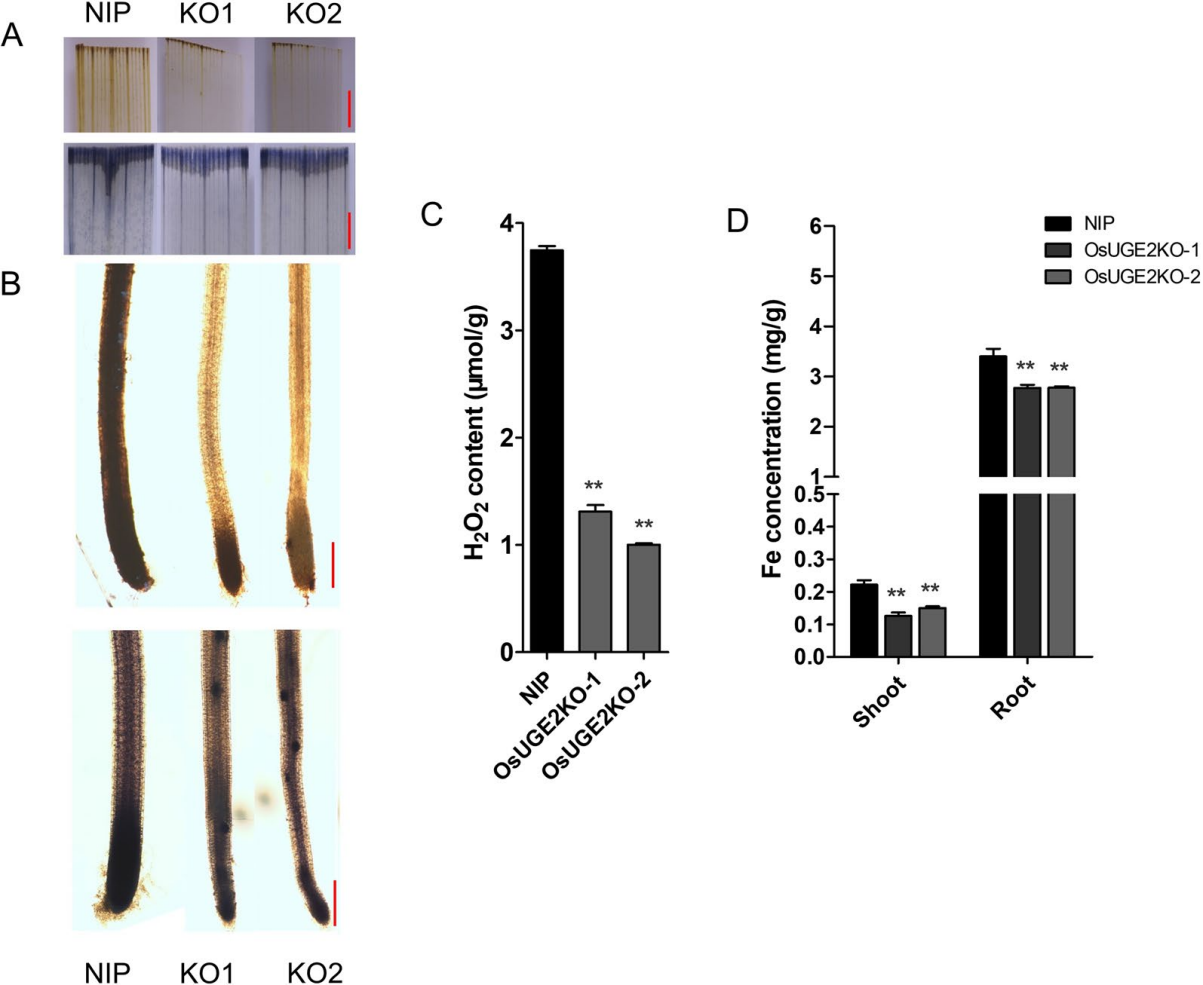
Knockout of *OsUGE2* decreases ROS and Fe contents. **A** DAB (up) and NBT (down) staining of leaves from 2-week-old seedlings cultivated in normal YNS hydroponic culture. Bars = 5 mm. **B** DAB (up) and NBT (down) staining of roots from 2-week-old seedlings grown in normal YNS hydroponic culture. Bars = 2 mm. **C** The content of H_2_O_2_ in 10-day-old rice seedlings cultivated in normal YNS hydroponic culture. The whole seedlings were sampled for measuring the H_2_O_2_ content. Three replications were used. **D** The content of Fe in dry shoot and root samples of 3-week-old seedlings grown in normal YNS hydroponic culture. Three replications were used. Data were means ± SEM. Asterisks indicated significant differences according to Student’s *t* test (*P* ≤ 0.05**)

To validate whether the content of ROS and Fe were influenced by loss of function of OsUGE2, DAB (3, 3′-diaminobenzidine) and NBT (nitroblue tetrazolium) staining were performed to detect the relative content of H_2_O_2_ and O_2_^−^. Twenty-day-old seedlings grown by hydroponics were used. The results showed that compared with NIP, both the DAB and NBT staining of leaves and roots of *OsUGE2KO-1* and *OsUGE2KO-2* lines were weaker (Fig. 7A, B), indicating reduced content of ROS in *OsUGE2* knockout mutants. To further confirm this result, the content of H_2_O_2_ in the whole plants of 10-day-old was measured. Indeed, consistence with the DAB staining, the H_2_O_2_ content of *OsUGE2KO-1* and *OsUGE2KO-2* lines was significantly decreased compared with NIP (Fig. 7C). Moreover, inductively coupled plasma mass spectrum (ICP-MS) was conducted to measure the Fe content. Twenty-day-old seedlings cultivated by the normal hydroponics were used. As shown in Fig. 7D, both in shoot and root, the Fe content in *OsUGE2KO-1* and *OsUGE2KO-2* lines was reduced notably. These results demonstrated that the ROS and Fe content in *OsUGE2* knockout mutants were decreased simultaneously.

### ***OsUGE2*** Knockout Mutants were Insensitive to Fe Deficiency and H_2_O_2_ Treatment

**Fig. 8:**
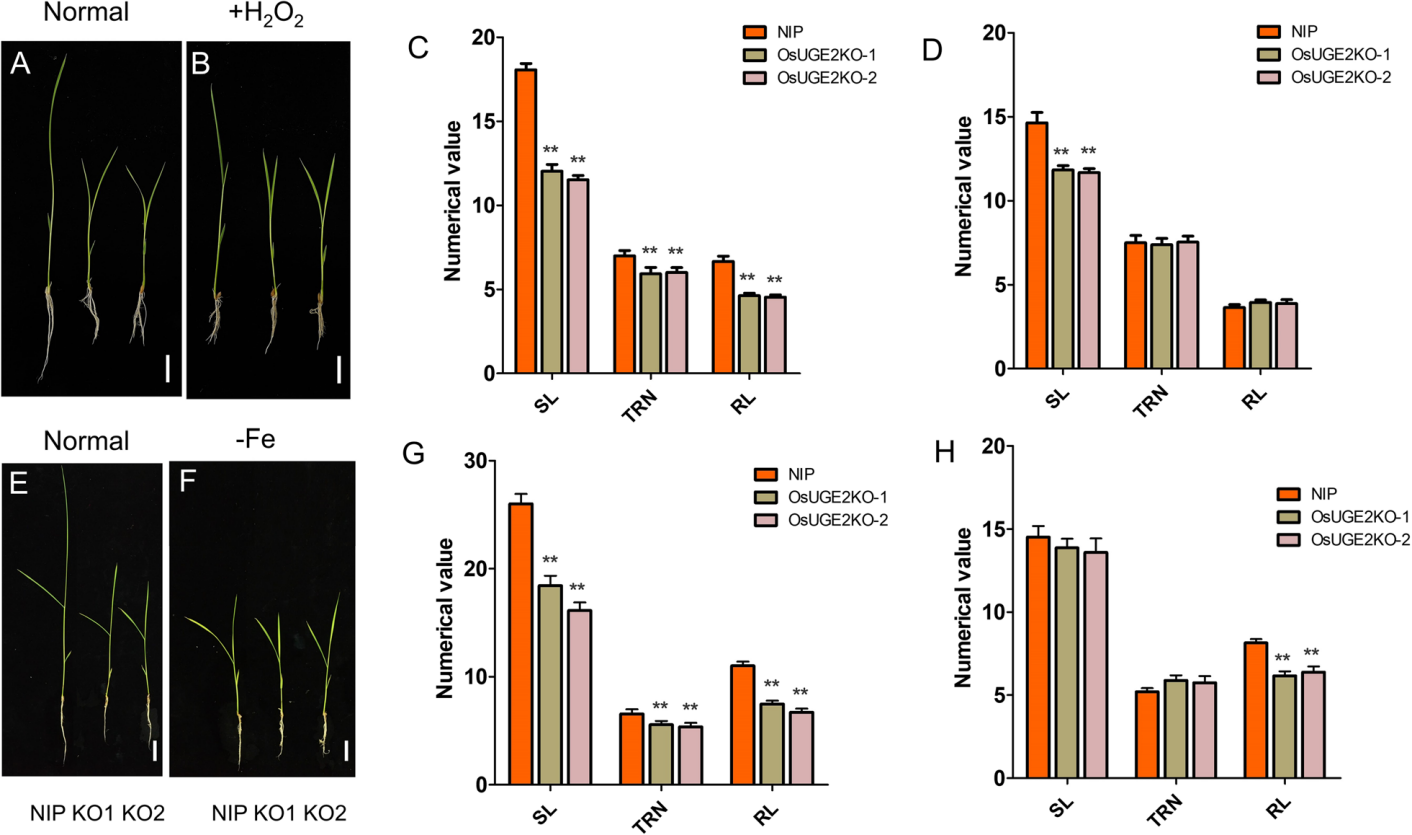
Knockout of *OsUGE2* was insensitive to -Fe treatment and H_2_O_2_ treatment. **A**, **B** Morphology of NIP, KO1 and KO2 grown in YNS hydroponic culture without (**A**) or with (**B**) H_2_O_2_ for 7 days. Bars = 2 cm. **C**, **D** Statistical analysis of SL, TRN and RL in NIP, KO1 and KO2 grown in YNS hydroponic culture without (**C**) or with (**D**) H_2_O_2_. The unit for SL and RL is centimeter (cm). At least 12 plants were measured for each genotype. **E**, **F** Morphology of NIP, KO1 and KO2 grown in normal YNS hydroponic culture (**E**) and Fe-deficiency hydroponic culture (**F**) for 14 days. Bars = 2 cm. **G**, **H** Statistical analysis of SL, TRN, RL under nutrient solution with (**G**) or without (**H**) Fe. The unit for SL and RL is centimeter (cm). At least 12 plants were measured for each genotype. Data were means ± SEM. Sterilized seeds were cultivated in ½ MS medium for 3 days under normal conditions and then transferred to the corresponding hydroponic culture. Asterisks indicated significant differences according to Student’s *t* test (*P* ≤ 0.05**)

Since loss-of-function of *OsUGE2* could decrease the content of H_2_O_2_ and Fe, we next investigated how is the response of *OsUGE2* knockout mutant to Fe deficiency and H_2_O_2_ treatments. Firstly, the response to H_2_O_2_ treatment was examined. The seeds of NIP and *OsUGE2* knockout mutants were germinated in half strength Murashige and Skoog (1/2 MS) medium for 3 days, then the rice seedlings were transferred to the hydroponic nutrient solution with or without H_2_O_2_ (0.5 mM). After cultivated for 7 days, the SL, TRN and RL were measured. As expected, knockout mutants were insensitive to H_2_O_2_ treatment compared with NIP (Fig. 8A–D). Specifically, the TRN and RL of knockout mutants were similar with those of NIP, and the SL of KO1 and KO2 was ~ 79.7% and ~ 80.8% of that in NIP under normal nutrient solution, while it was 90.9% and 92.7% under H_2_O_2_ treatment, respectively, although the difference was still significant (Fig. 8A–D).

For the response to Fe deficiency, seeds were also germinated in 1/2 MS medium for 3 days, then transferred to the hydroponic nutrient solution with or without Fe. After cultivated for 14 days, SL, TRN and RL were measured. Surprisingly, as shown in Fig. 8E–H, the knockout mutants appeared to be insensitive to Fe deficiency, which was contrary to our expectation. In detail, the SL and TRN of knockout mutants were similar to that of NIP, even the TRN showed a slight increasing trend under Fe deficiency (Fig. 8H). In normal nutrient solution, the RL of KO1 and KO2 was ~ 67.0% and ~ 60.2% of the RL of NIP, while under Fe deficiency, the ratio increased to ~ 78.3% and ~ 75.7%, respectively, which indicated that the RL of knockout mutants was also insensitive to the growth repression of Fe deficiency, although the difference between NIP and knockout mutants still remained significant (Fig. 8H). Previous study demonstrated that cell wall architecture is important for root apoplastic Fe reallocation (Peng et al. 2021), and OsUGE2 is involved in the cell wall composition and structure (Zhang et al. 2020a, b). This encouraged us to further investigate whether this insensitivity was related to the root apoplastic Fe reallocation. As expected, under Fe deficiency, the Fe content was not significantly different between NIP and *OsUGE2* knockout mutants in shoot and root, and knockout mutants displayed a slight increase of Fe content in shoot compared to that of NIP (Additional file 7: Fig. S7). This result implies that OsUGE2 might be involved in root apoplastic Fe reallocation and the apoplastic Fe pool of root in the *OsUGE2* knockout mutant seems to be more stable than that of NIP under Fe deficiency condition. Furthermore, the induced expression pattern was also examined. Interestingly, it seems that *OsUGE2* barely responded to Fe deficiency, which suggested that the regulation relationship between OsUGE2 and Fe level was single-direction (Additional file 8: Fig. S8). Collectively, these results clarified that H_2_O_2_ homeostasis and Fe level together controlled the growth in *OsUGE2* knockout mutants, at least partly.

## Discussion

In *Arabidopsis*, the *UGEs* gene family plays crucial roles in growth and development, and single or different *UGE* genes combinational mutants show severe growth and development deficiency (Rösti et al. 2007). In rice, previous studies demonstrated that *OsUGE2* and *OsUGE3* affect growth via changing the composition and structure of cell wall (Zhang et al. 2020a, b; Tang et al. 2022). However, the mechanism about how UGEs affecting growth still remains largely unknown. In this study, we propose that *OsUGE2* could retard rice growth and development at least partly through H_2_O_2_ and Fe homeostasis.

### Cell Wall Plays an Important Role in Maintaining Fe Homeostasis

The cell wall is mainly constituted of cellulose, hemicellulose and pectin, acting as the major component of apoplast and retaining a large number of cationic ions in plants (Carrier et al. 2003; Lei et al. 2014). Fe is considered to be difficult to remobilize, while the apoplastic Fe pool can be an essential Fe source during Fe deficiency period (Bienfait et al. 1985; Jin et al. 2007). It has been reported that more than 75% of Fe is stored in root apoplasts (Bienfait et al. 1985). Among the cell wall components, hemicellulose and pectin are proposed to mainly retain Fe (Xiong et al. 2009; Lei et al. 2014). Under Fe deficiency, some factors play important roles in the usage of the cell wall Fe pool. In *Arabidopsis*, Cdi can catalyze the transfer of GDP-L-galactose to the terminus of side chain A on RG-II, a type of pectin, and disruption of Cdi reduce RG-II dimerization, which weakens the reallocation of apoplastic Fe from roots to shoots due to disrupted cell wall (Peng et al. 2021). *ADC2-1*, a gene involved in polyamine putrescine (Put) biosynthesis, play an important role in reducing the Fe bound to root cell wall, especially to hemicellulose under Fe deficiency, thus increasing the soluble Fe content in root and shoot, consequently alleviating the Fe deficiency-induce chlorosis (Zhu et al. 2016). Abscisic acid (ABA) can also decrease Fe bound to pectin and hemicellulose to increase the shoot Fe content by promoting the secretion of phenolics to release apoplastic Fe (Lei et al. 2014). Therefore, cell wall indeed has an essential role in keeping Fe homeostasis in plants. *OsUGE2* was previously reported to alter the cell wall composition and structure by reducing accumulation of arabinogalactan, rhamnogalacturonan I and homogalacturonan (Zhang et al. 2020b), which indicates the Fe level in *OsUGE2* knockout mutant might be corresponding to the cell wall alteration. Under normal nutrient solution, the decreased Fe content may mainly result from the reduced expression of Fe related genes (Fig. 6D), while under Fe deficiency circumstance, the Fe content was similar with that in NIP (Additional file 7: Fig. S7), which might be due to the changed cell wall composition and structure. However, the mechanism is far from clear and still needs further investigation.

### The Retarded Growth in *OsUGE2* Knockout Mutant

ROS are important growth regulators in plants. Excessive or insufficient ROS are harmful to the growth and development of plants (Mittler 2017). However, basal ROS are necessary to maintain the normal growth and development (Lu et al. 2014; Schmidt and Schippers 2015; Mittler 2017). Iron is an indispensable nutrient element for plant growth and development and lack of iron will cause serious growth retardation in plants (Takahashi et al. 2001; Lee and An 2009; Yang et al. 2013). In our study, we demonstrated that loss of function of *OsUGE2* could decrease ROS content and Fe level probably through regulating the expression of genes related to oxidoreductase process and iron homeostasis, which finally led to the growth retardation of rice plants. Furthermore, it was reported that UDP-Gal is participated in galactolipid biosynthesis, which is the main component of chloroplasts (Li et al. 2011). In our study, loss of function of *OsUGE2* not only increased the ratio of UDP-Glc/UDP-Gal, but also decreased the content of both UDP-Glc and UDP-Gal (Fig. 3), and the latter may be due to reduced photosynthesis ability (Additional file 5: Fig. S5B to E) because of aberrant chloroplasts development (Zhang et al. 2020a) and decreased pigment content (Additional file 5: Fig. S5A). Iron is known to alter chloroplast structure and photosynthesis rate in higher plants (Eberhard et al. 2008). The deficiency of Fe also changes chlorophyll synthesis (Tottey et al. 2003). Therefore, the weakened photosynthesis ability should result from both decreased UDP-Gal and Fe level, which together contribute to the growth retardation of *OsUGE2* knockout mutant. Moreover, photosynthesis is one of the main ways to produce ROS (Huang et al. 2016). Thus, the reduced photosynthesis of *OsUGE2* knockout mutant may be one of the reasons for decreased ROS content (Fig. 7A–C), which also possibly leads to retarded growth. However, the growth retardation of *OsUGE2* knockout mutant is sophisticated, but at least, Fe and ROS were the two contributors for growth retardation.

### The Possible Reasons for Gene Expression Alteration in *OsUGE2* Knockout Mutant

It is amazing that how a single enzyme caused expression changes of such huge amount of genes in *OsUGE2* knockout mutant (Fig. 6A). In plants, UDP-Glc/Gal are the key intermediate substrates for synthesis of starch, cellulose, hemicellulose, pectin and other types of sugars (González-Morales et al. 2016; Wang et al. 2021). However, in addition to as substrates, UDP-Glc/Gal may also have the possibility to act as a signal molecule, which induces the production of ROS in rice (Chen et al. 2023). In animal model, UDP-Glc and other UDP-sugars have been proved to bind with P2Y_14_R, a G protein-coupled receptor (GPCR), further affecting the initiation and progression of downstream responses (Karcz et al. 2021). In plants, the regulator of G protein signaling-1 (RGS1) protein is a membrane receptor for D-glucose, which is important for glucose-sensing signal transduction pathways to control growth and development (Johnston et al. 2007; Wang et al. 2022d). Furthermore, growing evidences show that sucrose, fructose, and trehalose-6-phosphate also function as signaling molecules to regulate plant growth and development (Chen et al. 2022). Therefore, the disrupted homeostasis of UDP-Glc/Gal themselves, or the products of them, which acting as a signal molecule, may result in the gene expression differences at the whole genome-wide level between NIP and *OsUGE2* knockout mutant, including the genes related to the hydrogen peroxide catabolic and metabolic process and iron ion homeostasis Another possible explanation for the expression changes may be due to the alteration of cell wall. *Osuge2* mutant displays reduced arabinogalactan proteins (AGP), pectins and cellulose (Zhang et al. 2020a, b). Of them, AGP could bind to hemicellulose and pectins to provide adhesive or positional cues (Chebli et al. 2021). It has been suggested that cell wall integrity (CWI) signaling pathway play important roles in growth and development in plants, and some studies demonstrated that wall-associated kinases (WAKs) can act as CWI receptors, which are involved in cell elongation and the coordination of solute concentration with growth (Kohorn and Kohorn 2012; Wolf et al. 2012). Consistence with this, we also found that in *OsUGE2* knockout mutant, the expression of some *OsWAKs* were changed significantly (Additional file 9: Fig. S9), which was likely to contribute to the expression alteration of downstream genes containing the genes responsible for the oxidoreductase process and iron homeostasis. Even more, a most recent report demonstrated that OsUGE1, a homology protein of OsUGE2, could play as a transcription factor (Wang et al. 2023), which indicated that OsUGE2 may also act as a transcription factor to regulate the gene transcription level directly or indirectly, involved with the genes regulating the ROS level and iron homeostasis.

## Conclusions

In this study, we revealed that knockout of *OsUGE2* significantly altered the ratio and content of UDP-Glc and UDP-Gal, which further caused decreased ROS and Fe content by influencing the expression level of genes related to hydrogen peroxide catabolic and metabolic process and iron ion homeostasis, finally resulting in retarded growth through reduced cell length.

## Materials and Methods

### Plant Materials and Growth Conditions

The transgenic materials in this study were in Nipponbare (NIP) background. For knockout mutants, the CRISPR-Cas9 technology was applied (Ma et al. 2015a), and the target sequence for *OsUGE2* was 5′ CCATTGCTTTATTACGACAACAA 3′. For *pro*_*OsUGE2*_*::GUS* lines, 3207 bp of upstream of initiation codon was amplified and cloned into *pCAMBIA1301* vector fused with GUS reporter. Calli induced from NIP seeds was used for transformation with *Agrobacterium tumefaciens* EHA105 carrying the related plasmids. The target sequences for *OsUGE1*, *OsUGE3* and *OsUGE4* and the related primers were listed in Additional file 10: Table S1.

Rice seeds were sterilized with 75% ethanol for 1 min and 2.5% sodium hypochlorite for 15 min in sequence, followed by 3–5 times rinses with sterile distilled water. Then the seeds were placed on 1/2 Murashige and Skoog (MS) medium with agar for germination. The hydroponic culture was the Yoshida nutrient solution (YNS) (Impa et al. 2013). The growth conditions were 30 °C light/28 °C dark (14 h/10 h) with 60–70% relative humidity. Under natural environment, rice plants were grown in pots with soil derived from paddy field.

### Phenotype Observation

Rice plants of different growth stages were measured or counted. The visible length was measured with a ruler. For root length measurement, the longest root length was counted as root length. For measurements of cell length, 3-week-old rice seedlings cultivated in hydroponic culture were used. Inner-epidermal of leaf sheath were staining with 0.5% toluidine blue. The cross and longitudinal sections were obtained by paraffin section method as reported previously (Zhao et al. 2021). Images were captured by a light microscope (Nikon ECLPSE Ni, Japan) and post-processed with Image J software and Adobe Photoshop CC software.

### DNA Extraction and PCR Analysis

Rice genomic DNA was extracted through modified CTAB (Hexadecyltrimethy Ammounium Bromide) method (Murray and Thompson 1980) with fresh leaves obtained from young plants. PCR reaction system was as follows: denaturation at 94 °C for 5 min; then running for 28–32 cycles with 94 °C for 30 s, 55 °C for 30 s, 72 °C for 60–180 s; and the final step was extension for 5 min. For identification of positive plants, the products of PCR from transgenic plants genomic were sequenced by Sanger sequencing technology, then genotyping was analyzed with the DSD method as previously reported (Ma et al. 2015b). The related primers were listed in Additional file 10: Table S1.

### RNA Isolation and Gene Expression Analysis

Total RNA was extracted using RNAiso™ Plus (Takara, Japan). First-strand cDNA was synthesized from 1 μg total RNA by the Evo M-MLV reverse transcriptase kit (Accurate Biology, China). The qRT-PCR assays were performed using the SYBR® Green Pro Taq HS qPCR kit (Accurate Biology, China) in a Roche LightCycler®96 system. The PCR procedures were as follows: 95 °C for 30 s, then followed by 40 cycles of 95 °C for 5 s, 60 °C for 30 s, and the final step was dissociation stage. *OsActin* was used as an internal control and the data analysis was referred to 2^−△△Ct^ algorithm. Three biological replicates were used and the related primers were listed in Additional file 10: Table S1.

### Subcellular Localization and Histochemical GUS Staining

The full-length cDNA of *OsUGE2* was amplified and cloned into *p35S-GFP* vector. The rice protoplasts were extracted from the leaf sheaths derived from 10-day-old seedlings cultivated in hydroponic culture according to the method reported previously (Zhang et al. 2011). Afterward, the fused vector was transformed into rice protoplasts by a PEG-mediated method (Zhang et al. 2011). After incubating overnight, the fluorescence signals were detected by a FV31S-SW confocal microscope (OLYMPUS, Japan).

For GUS staining, tissues were collected from *pro*_*OsUGE2*_*::GUS* lines and incubated at 37 °C for 12 h with staining buffer referred to the method reported previously (Li et al. 2017). Then the tissues were decolorized in 75% ethanol. The stained tissues were photographed by a stereo microscope (Nikon AZ100, Japan). The related primers were listed in Additional file 10: Table S1.

### ROS Assays

For ROS staining, the fresh leaves and roots of 18-day-old seedlings cultivated in hydroponic culture were used. Nitroblue tetrazolium (NBT) and 3,3′-diaminobenzidine (DAB) were applied to stain the above tissues to detect O_2_^−^ and H_2_O_2_, respectively. The staining method was referred to the report described previously (Zhao et al. 2021). For the quantitative analysis of H_2_O_2_, 10-days-old rice seedlings cultivated by hydroponic culture were used. The content of H_2_O_2_ was measured by a hydrogen peroxide detection kit (Cat No. BC3590, Solarbio, China) and performed according to the instructions. About 100 mg fresh samples were grinded into powder in liquid nitrogen and added 1 mL ice-cold reagent I to homogeny, followed by centrifuging at 8000*g*, 4 °C for 10 min. After that, all the supernatant was taken and added various hydrogen peroxide detection reagents as the instructions described. Absorbance value at a wavelength of 415 nm was measured for the final reaction solution, and the calculation of H_2_O_2_ content was performed according to the formula described in the instructions.

### RNA-Sequencing (RNA-seq) Analysis

For RNA-seq analysis, 3-week-old seedlings cultivated by normal YNS hydroponic culture were used and the growth conditions were described as above. Total RNA extracted from the whole rice seedlings of NIP and *OsUGE2KO-1* line was used for RNA-seq analysis with three independent biological replications. The cDNA libraries were generated and sequenced on the DNBSEQ-T7RS platform (Gene^+^, Shenzhen, China) with PE150 strategy. The clean reads were aligned to the NIP genome sequence (IRGSP-1.0) in National Center for Biotechnology Information (NCBI) database. The differentially expressed genes (DEGs) (*P*_adj_ ≤ 0.05 and fold change ≥ 2) were screened by DESeq2 software. GO enrichment analysis was performed by g: Profiler software and the DEGs used for heat map were selected according to the GO enrichment analysis. TBtools software (Chen et al. 2020) was used to draw the GO enrichment map and heat maps. For the heat maps, the fragments per kilo base per million mapped reads (FPKM) were used and calculated with log_2_^(FPKM)^. Row scale method selected Zero To One. The specific analysis method was referred to the previous report (Chen et al. 2020).

### Measurement of UDP-Glc and UDP-Gal Content

The shoots of 1-week-old seedlings grown under normal YNS hydroponic culture were used for measuring the content of UDP-Glc and UDP-Gal. The extraction method was referred to the report described previously (Behmüller et al. 2014). Briefly, samples about 40 mg was ground into fine powder in liquid nitrogen and extracted with 600 µl ice-cold chloroform–methanol (3:7, v/v) at below 20 °C for 2 h. After that, 400 µl ddH_2_O was added to the sample and vortexed thoroughly, followed by centrifugation for 5 min at 20,000*g* under 4 °C. The upper clear phase was collected and repeated the above two steps for another two times. Then the upper clear phase mixed together and freeze-dried for 4 h. Finally, the dry matter was suspended in 200 µl ddH_2_O and ready for detection. The UDP-Glc and UDP-Gal standards was purchased from Macklin Biochemical Technology Co. Ltd. (Shanghai, China). The nucleotide sugar detection was performed by liquid chromatography-tandem mass spectrometry (LC–MS/MS) using a 4000 QTRAP LC–MS/MS system (Sciex, CA) and an Agilent 1100 Series Capillary LC System (Agilent Technologies, USA), as described previously (Rautengarten et al. 2016).

### Measurement of Fe Content

For Fe content analysis, dry samples of shoots and roots harvested from rice seedlings grown in both Fe-sufficient and Fe-deficient conditions were digested in 2 ml concentrated nitric acid for 5 h at 135 °C. Then the samples were detected by the inductively coupled plasma mass spectrometry (ICP-MS) system (Agilent 7500ce, USA).

### Measurement of Chlorophyll Content and Photosynthetic Rate

Rice leaves of 2-week-old plants were used for pigments measurement. The chlorophyll was extracted by 95% ethanol and detected the OD value at 665 and 649 nm, respectively. The calculation was referred to the method described previously (Sartory and Grobbelaar 1984). For the determination of photosynthetic rate, intercellular CO_2_ concentration, stomatal conductance and transpiration rate, the uppermost fully expanded leaves of 2-month-old rice plants were selected and measured by a LI-6400 portable photosynthesis system (LI-CRO, USA) according to the manufacturer’s instructions.

### Statistical Analysis

Statistical analysis was performed using the software GraphPad Prism 5.0 with Student’s *t* test. Significant differences were evaluated at *P* ≤ 0.05**.

### Supplementary Information

**Additional file 1**. Schematic representation of knockout targets’ location of *OsUGE2*.

**Additional file 2**. Mutation sites of *OsUGE2* knockout mutants.

**Additional file3**. Simultaneous mutation of all the *OsUGEs* gene family severely retarded the rice growth.

**Additional file 4**. *OsUGE2* affected the expression of* OsUGE3* and *OsUGE4*.

**Additional file 5**. Photosynthetic rate is significantly decreased in *OsUGE2* knockout mutant. 

**Additional file 6**. qRT-PCR identification of RNA-seq results.

**Additional file 7**. Fe content of NIP, *OsUGE2KO-1* and *OsUGE2KO-2* grown under Fe-deficiency condition.

**Additional file 8**. *OsUGE2* is barely induced by -Fe treatment.

**Additional file 9**. Heat map of DEGs clustering related to wall-associated kinases (WAKs) based on RNA-seq analysis.

**Additional file 10**. The primers used in this article.

## Data Availability

The raw RNA-seq data of this paper has been deposited in the NCBI Sequence Read Archive under the Bioproject accession number PRJNA895101, and the metadata is available at https://dataview.ncbi.nlm.nih.gov/object/PRJNA895101?reviewer=labph8qc91kl8u6k6lgeh1nsvh.

## Abbreviations

ROS: Reactive oxygen species
DAB: 3,3′-Diaminobenzidine
NBT: Nitroblue tetrazolium
UDP-Glc: UDP-Glucose
UDP-Gal: UDP-Galactose
ICP-MS: Inductively coupled plasma mass spectrometry
LC–MS: Liquid chromatography–tandem mass spectrometry
DEGs: Differentially expressed genes
CTAB: Hexadecyltrimethy Ammounium Bromide
SL: Shoot length
TRN: Total root number
RL: Root length
