# Supplementary figures and images for: OsUGE2 Regulates Plant Growth through Affecting ROS Homeostasis and Iron Level in Rice
Source: Rice (N Y). 2024 Jan 12;17:6. doi: 10.1186/s12284-024-00685-0 (PMC10784444; doi:10.1186/s12284-024-00685-0)

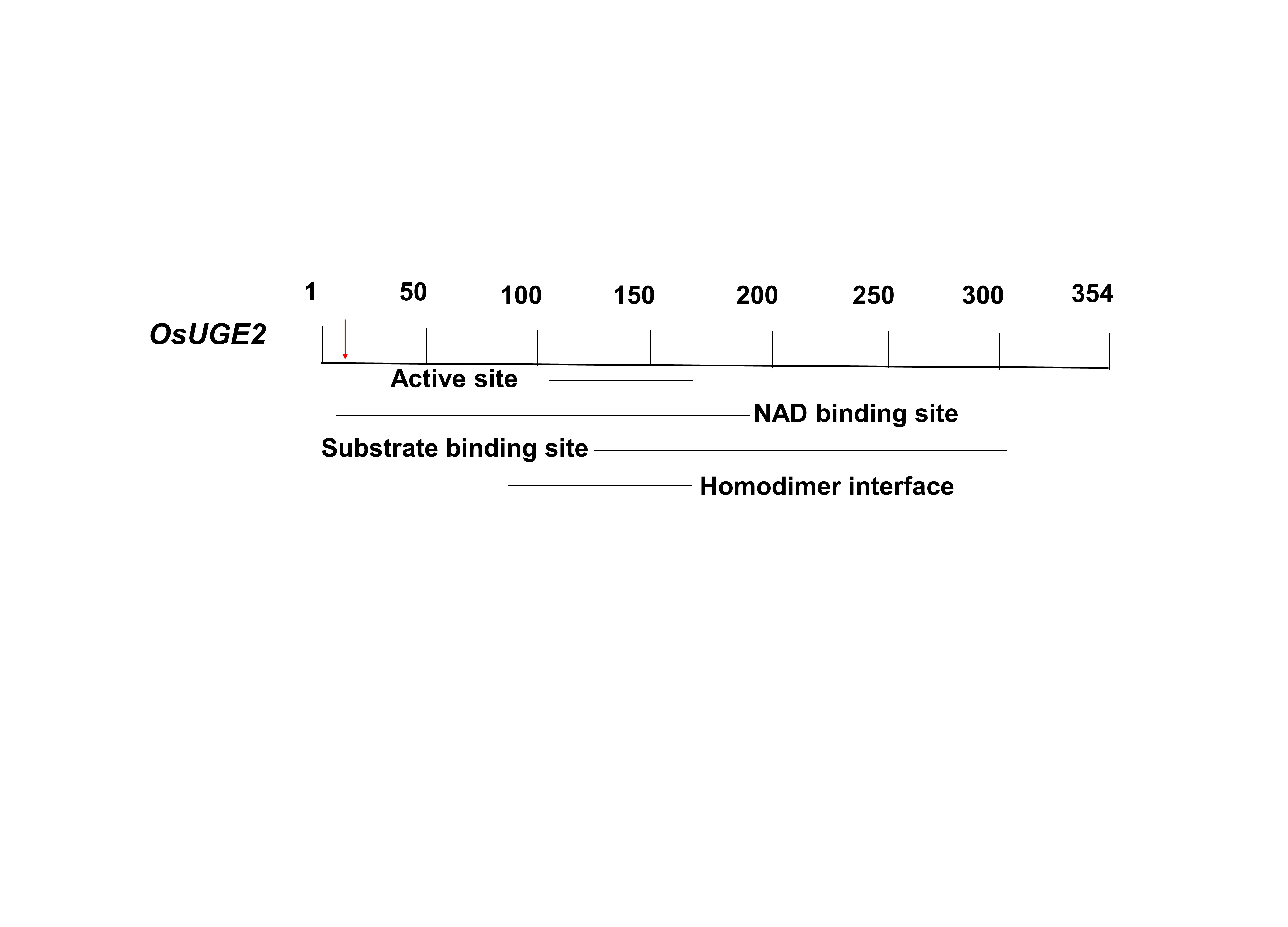

Supplement: Supplementary file 1 — Additional file 1. Schematic representation of knockout targets’ location of OsUGE2. [file 12284_2024_685_MOESM1_ESM.jpg]

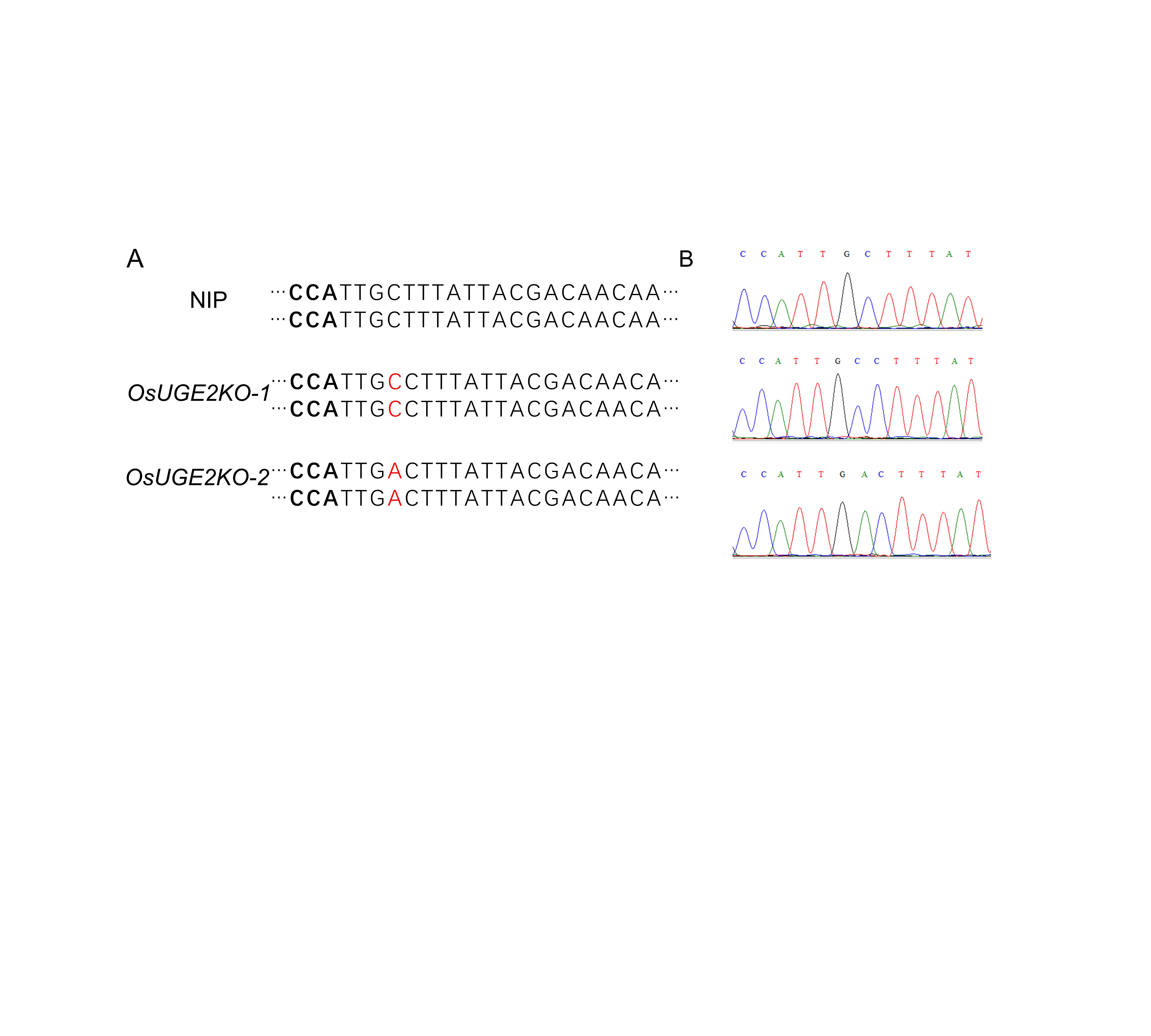

Supplement: Supplementary file 2 — Additional file 2. Mutation sites of OsUGE2 knockout mutants. [file 12284_2024_685_MOESM2_ESM.jpg]

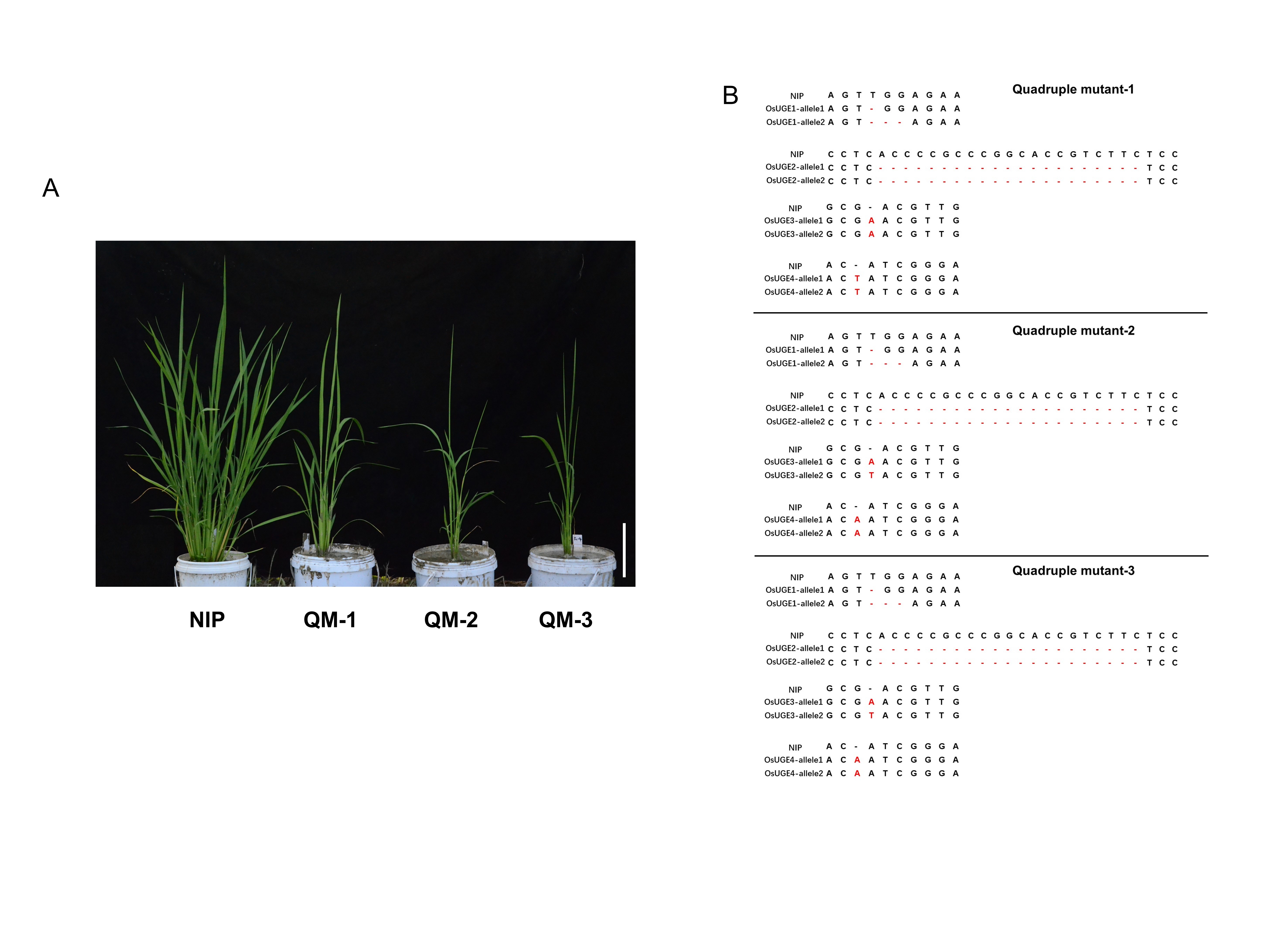

Supplement: Supplementary file 3 — Additional file3. Simultaneous mutation of all the OsUGEs gene family severely retarded the rice growth. [file 12284_2024_685_MOESM3_ESM.jpg]

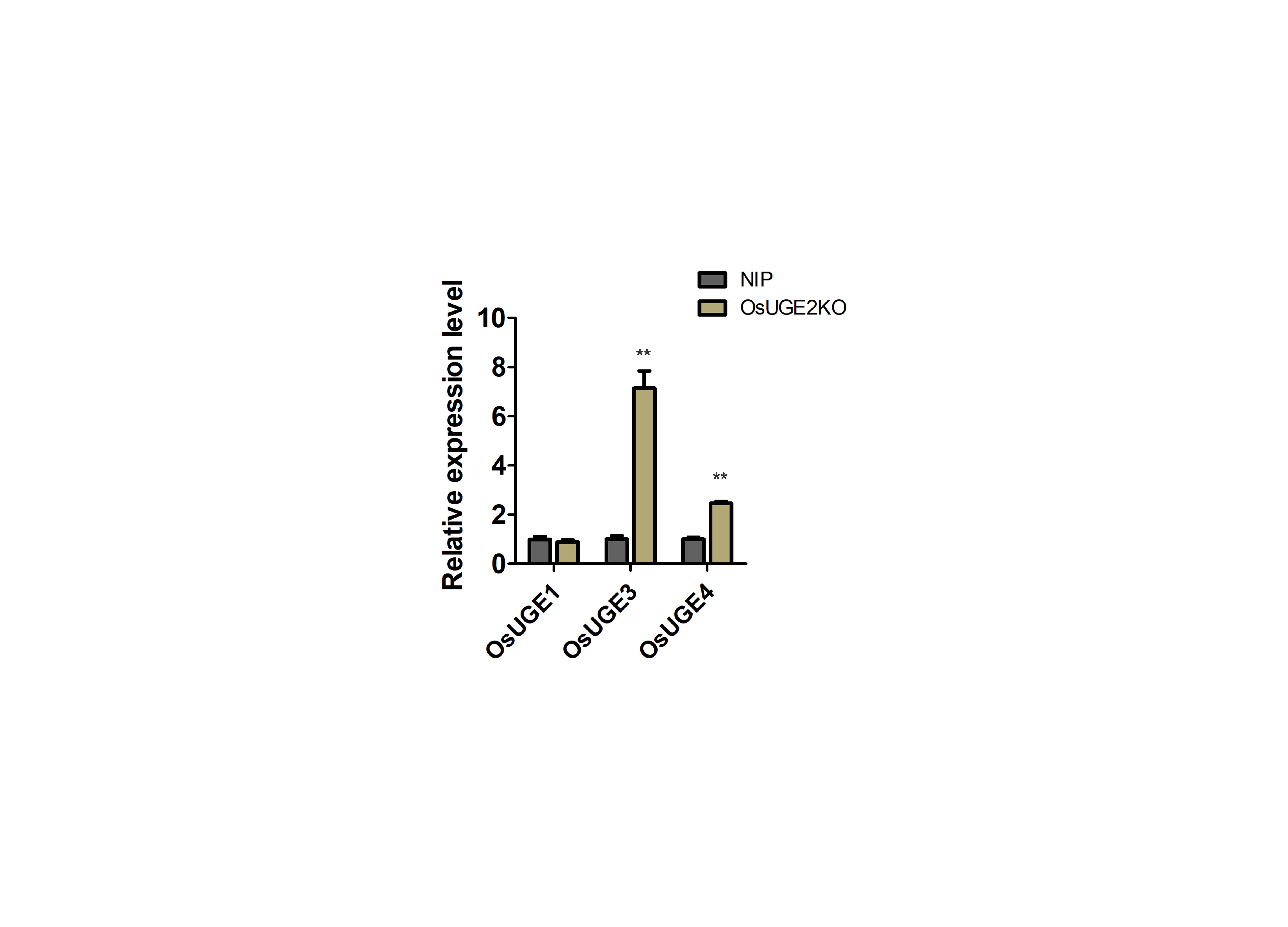

Supplement: Supplementary file 4 — Additional file 4. OsUGE2 affected the expression of OsUGE3 and OsUGE4. [file 12284_2024_685_MOESM4_ESM.jpg]

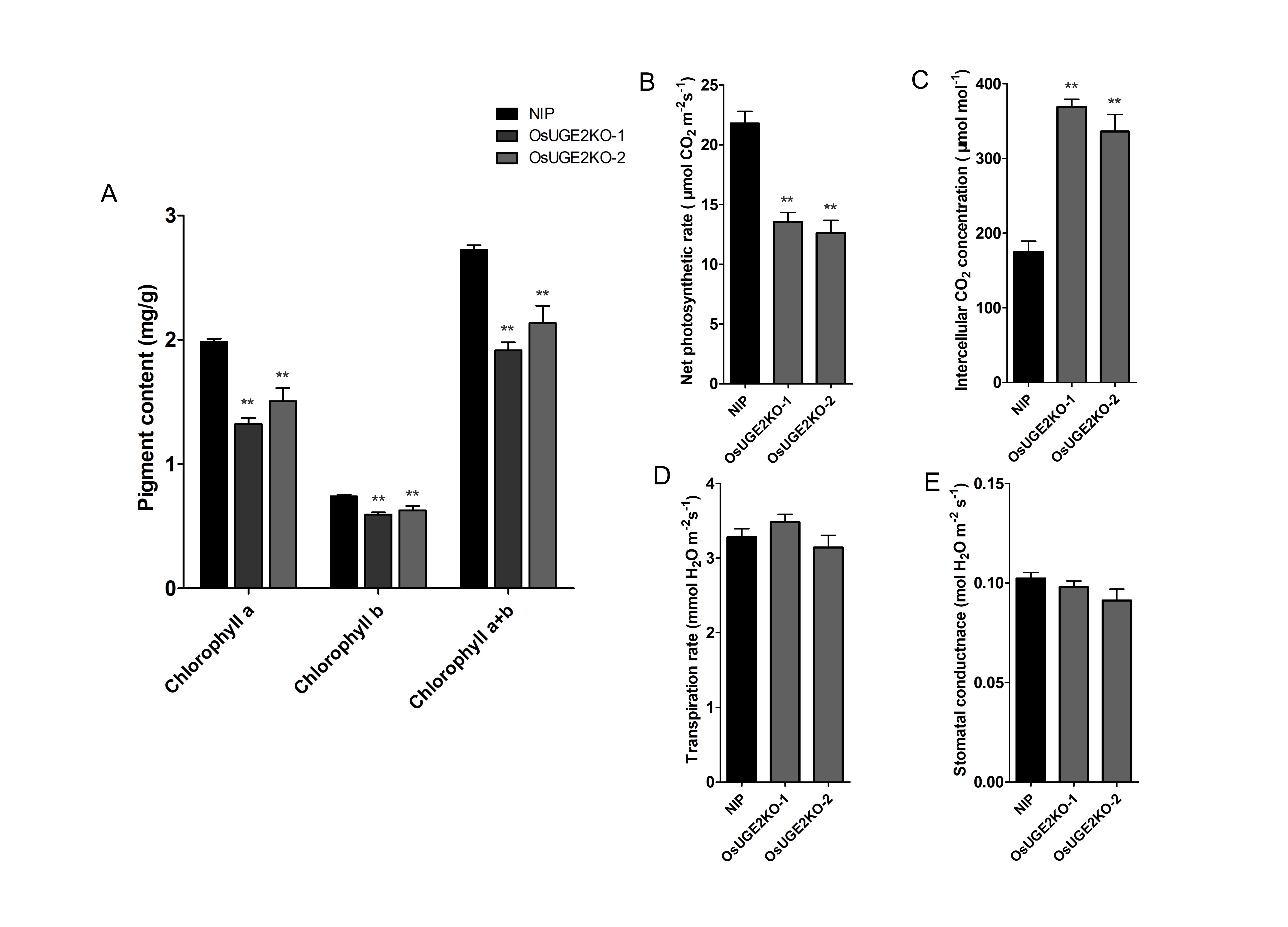

Supplement: Supplementary file 5 — Additional file 5. Photosynthetic rate is significantly decreased in OsUGE2 knockout mutant. [file 12284_2024_685_MOESM5_ESM.jpg]

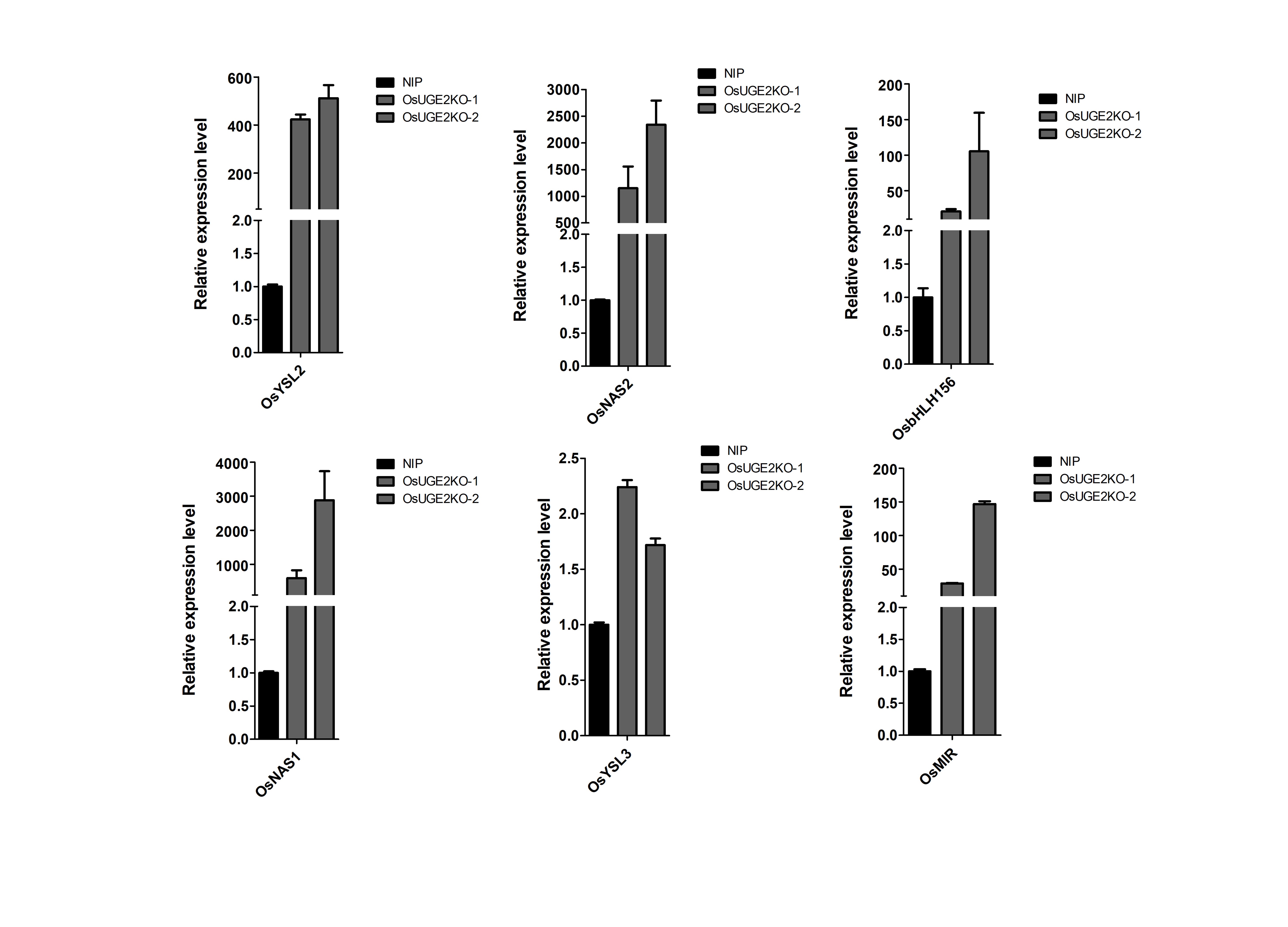

Supplement: Supplementary file 6 — Additional file 6. qRT-PCR identification of RNA-seq results. [file 12284_2024_685_MOESM6_ESM.jpg]

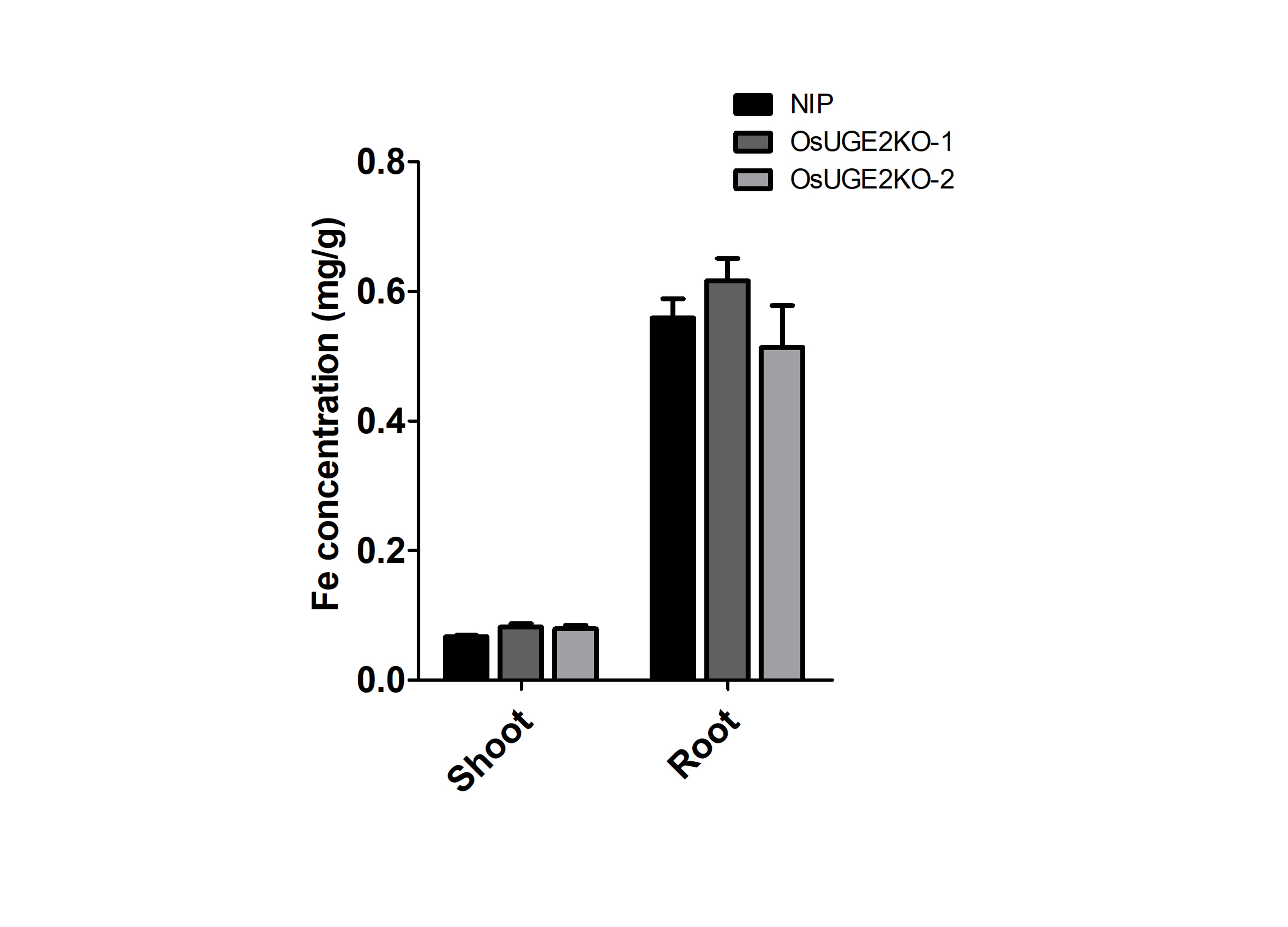

Supplement: Supplementary file 7 — Additional file 7. Fe content of NIP, OsUGE2KO-1 and OsUGE2KO-2 grown under Fe-deficiency condition. [file 12284_2024_685_MOESM7_ESM.jpg]

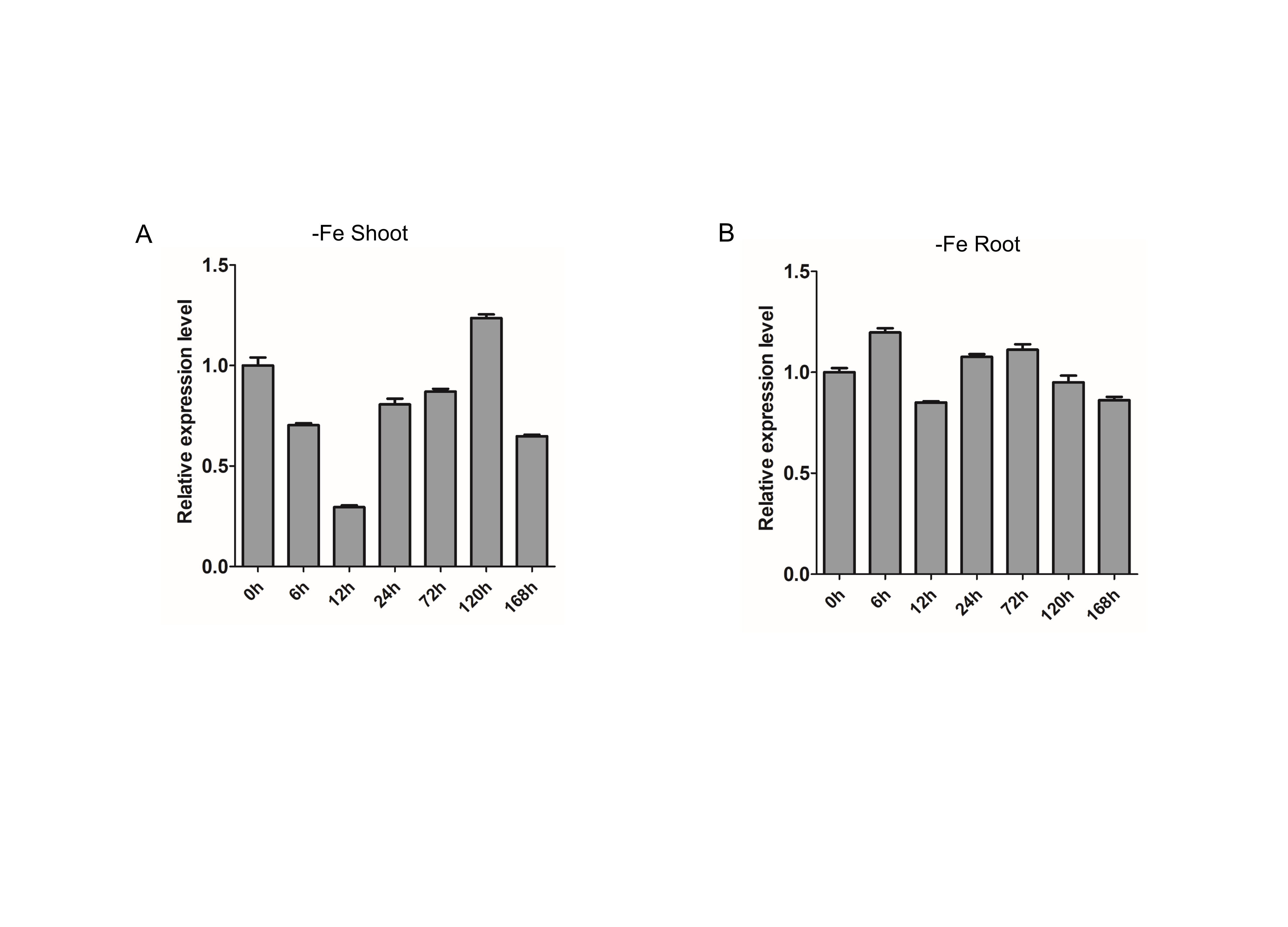

Supplement: Supplementary file 8 — Additional file 8. OsUGE2 is barely induced by -Fe treatment. [file 12284_2024_685_MOESM8_ESM.jpg]

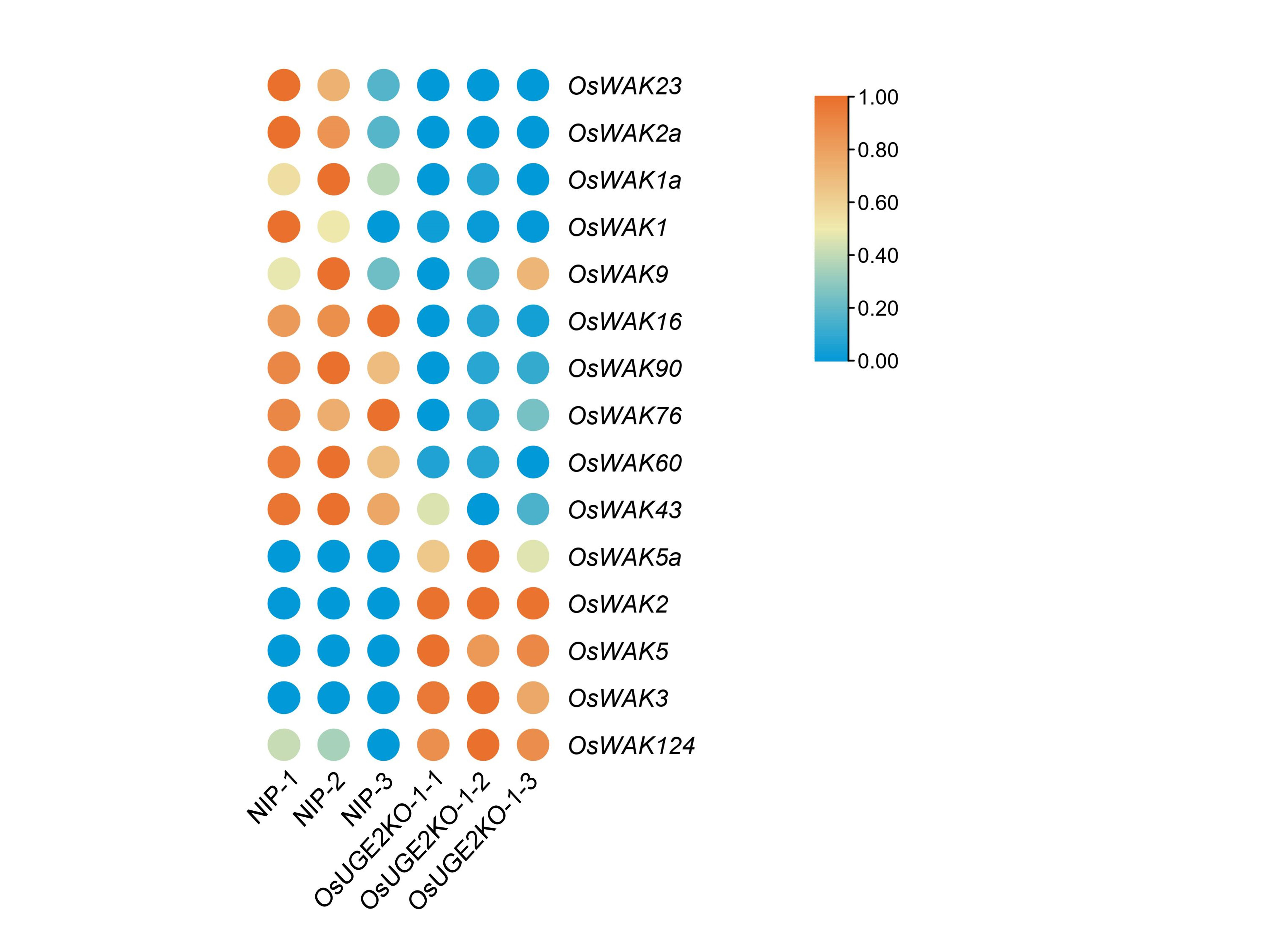

Supplement: Supplementary file 9 — Additional file 9. Heat map of DEGs clustering related to wall-associated kinases (WAKs) based on RNA-seq analysis. [file 12284_2024_685_MOESM9_ESM.jpg]
