# Supplementary material for: OsUGE2 Regulates Plant Growth through Affecting ROS Homeostasis and Iron Level in Rice
Source: Rice (N Y). 2024 Jan 12;17:6. doi: 10.1186/s12284-024-00685-0 (PMC10784444; doi:10.1186/s12284-024-00685-0)
Supplement: Supplementary file 10 — Additional file 10. The primers used in this article. [file 12284_2024_685_MOESM10_ESM.docx]

**Supporting information**

**Figure S1. Schematic representation of knockout targets’ location of *OsUGE2***.

The target location of OsUGE2 indicated by red arrow. The black lines indicated the relative location of conservative sites, including active site, NAD binding site, substrate binding site and homodimer interface.

**Figure S2. Mutation sites of *OsUGE2* knockout mutants.**

**(A)** Mutation sites of *OsUGE2KO-1* and *OsUGE2KO-2* generated by CRISPR-Cas9 system. The red character mean single-base insertion.

**(B)** Sequencing chromatogram of the edition sites, which were corresponding to NIP, *OsUGE2KO-*1 and *OsUGE2KO-2* from top to bottom.

**Figure S3. Simultaneous mutation of all the *OsUGEs* gene family severely retarded the rice growth.**

**(A)** Morphology of quadruple mutant of *OsUGEs* gene family at tillering stage. Bars=10cm. **(B)** Mutation sites of three single plants of quadruple mutant generated by CRISPR-Cas9 system. The single red line mean single-base deletion and the red character mean single-base insertion.

**Figure S4. *OsUGE2* affected the expression of *OsUGE3* and *OsUGE4*.**

*OsActin* was used as an internal control. Total RNA extracted from leaves of 10-day-old *OsUGE2KO-1* line was used for analysis. The expression level in NIP was set as “1”. Three biological replications were used. Data were means ± SEM. Asterisks indicated significant differences according to Student’s *t*-test (P≤0.05**).

**Figure S5. Photosynthetic rate is significantly decreased in *OsUGE2* knockout mutant.**

**(A)** The pigment content in NIP, *OsUGE2KO-1* and *OsUGE2KO-2*. Leaves of two-week old rice plants cultivated in normal YSN hydroponic culture were sampled for pigments measurement. Three replications were used.

**(B to E)** Net photosynthetic rate (B), intercellular CO2 concentration (C), transpiration rate **(D)** and stomatal conductance (E) in NIP, *OsUGE2KO-1* and *OsUGE2KO-2*. Two-month-old rice plants were used. At least eight plants were measured for each genotype.

Data were means ± SEM. Asterisks indicated significant differences according to Student’s *t*-test (P≤0.05**).

**Figure S6. qRT-PCR identification of RNA-seq results.**

The expression level of genes in NIP was set as “1”, the bar chart showed the downregulated times of genes compared with those of NIP. *OsActin* was used as an internal control. Total RNA extracted from the whole three-week-old rice seedlings was used for analysis. Three biological replications were used. Data were means ± SEM.

**Figure S7. Fe content of NIP, *OsUGE2KO-1* and *OsUGE2KO-2* grown under Fe-deficiency condition.** Sterilized seeds were cultivated in ½ MS medium for 3 days under normal conditions and then transferred to the Fe-deficiency hydroponic culture for three weeks. Dry shoot and root samples were used for Fe content measurement with three replications. Data were means ± SEM.

**Figure S8. *OsUGE2* is barely induced by -Fe treatment.**

**(A to B)** The induced expression profile of *OsUGE2* in shoot (A) and root (B) of NIP plants. Two-week-old seedlings grown in normal nutrient solution were transferred to the Fe-deficiency nutrient solution and sampled at different times. Three biological replications were used. Data were means ± SEM.

**Figure S9. Heat map of DEGs clustering related to wall-associated kinases (WAKs) based on RNA-seq analysis.**

**Table S1. The primers used in this article.**

| Knockout target sequences for quadruple mutant | |
| --- | --- |
| *OsUGE1* | TCCTCCAGCTTCTCCAACT |
| *OsUGE2* | GGCGGGGTGAGGACGGTGCT |
| *OsUGE3* | TACTACGAGAACAACGTCGC |
| *OsUGE4* | CGGGAGGGGCCGGGTACATC |
| Identification of positive transgenic plants | |
| JD-*OsUGE1KO*-F | GAAGCTAGGAAAAAGGGAAATA |
| JD-*OsUGE1KO*-R | TCGCCCGAATACAGATATAAGT |
| JD-*OsUGE2KO*-F | CTCCTCCCCACATTCCCAAAGAGAA |
| JD-*OsUGE2KO*-R | CATTGCACGGAGAGGGAGAGAT |
| JD-*OsUGE3KO*-F | GATACGGGTCAAAAAGGGCACGG |
| JD-*OsUGE3KO*-R | TCAGCTTGGAATCCTCGACGCAG |
| JD-*OsUGE4KO*-F | TACTAATCCCACAAGATGCCGA |
| JD-*OsUGE4KO*-R | TTAGTGTGTGTAGCAAGGGTGG |
| JD-*OsUGE1GUS*-F | CTGCTCTAGCCAATACGCAAACC |
| JD-*OsUGE1GUS*-R | CTCATCCGCAATCTATCCAACCA |
| Amplification of full length cDNA | |
| *OsUGE2*-F | GTGGTGGTGCTTCTGGTCTTG |
| *OsUGE2*-R | CACAGGCAGTGGGATTCTTCA |
| Subcellular localization | |
| *OsUGE2-GFP*-F | ACGGGGGACTCTAGAGGATCCATGGCGGTGGAGAAGACGGTG |
| *OsUGE2-GFP*-R | GCCCTTGCTCACCATGGTACCGTGGAAGTGGCCGTTGCCGTTAT |
| Amplification of OsUGE1 promoter | |
| *Pro_OsUGE2_*-F | CTATGACATGATTACGAATTCACACCCTCTGATATACAGCATTACA |
| *Pro_OsUGE2_*-R | TTACCCTCAGATCTACCATGGCTCTCTCTCTCTCACACACACACACCTC |
| Quantitative RT-PCR analysis | |
| qRT-*OsActin*-F | ACCATTGGTGCTGAGCGTTT |
| qRT-*OsActin*-R | CGCAGCTTCCATTCCTATGAA |
| qRT-*OsUGE1*-F | GTTTGAGGCTGTCATCCATTTT |
| qRT-*OsUGE1*-R | GTTGTTGTCGTAGTAAAGCAGG |
| qRT-*OsUGE2*-F | GAACTCAACTGGAAAGCGAAAT |
| qRT-*OsUGE2*-R | TCAAGTGACCCGTATCCATAAG |
| qRT-*OsUGE3*-F | CAACAACCTTCTTCCGTACATC |
| qRT-*OsUGE3*-R | GATTGTAAGCCACACAACCAAT |
| qRT-*OsUGE4*-F | TGGTGATGCTGAGATTCTCTTT |
| qRT-*OsUGE4*-R | GCACATTTCTTCGATGCCATAT |
| qRT-*OsYSL2*-F | AGCGACAGATGAGGATACATTT |
| qRT-*OsYSL2*-R | GTTGGAACATTATGGGTATCGC |
| qRT-*OsNAS2*-F | CATAGTAATCCTGGCTGTGTCT |
| qRT-*OsNAS2*-R | GAAGCACAAACACAAACCGATA |
| qRT-*OsbHLH156*-F | TGCAAGAGTGGCAGAATAATTG |
| qRT-*OsbHLH156*-R | GTAGAGCTTCTCCTTCATCCG |
| qRT-*OsNAS1*-F | GTTGAGAAGGCAGAAGAGTTTG |
| qRT-*OsNAS1*-R | CCATAATATAGTGCGCCTTTCG |
| qRT-*OsYSL3*-F | TGTTTAGTTGCCTGTCTCATCT |
| qRT-*OsYSL3*-R | CAAAAAGGACACACGATCAACT |
| qRT-*OsMIR*-F | GCTTCTCTTTGTGTTGACTGTT |
| qRT-*OsMIR*-R | AGTGCTCAACTGTATATGACGT |
